# Resolving the Nanostructure of Carbon Nitride‐Supported Single‐Atom Catalysts

**DOI:** 10.1002/smll.202408286

**Published:** 2025-01-09

**Authors:** Nicolò Allasia, Shuai Xu, Sadaf Fatima Jafri, Elisa Borfecchia, Luis A. Cipriano, Giancarlo Terraneo, Sergio Tosoni, Lorenzo Mino, Giovanni Di Liberto, Gianfranco Pacchioni, Gianvito Vilé

**Affiliations:** ^1^ Department of Chemistry, Materials and Chemical Engineering “Giulio Natta” Politecnico di Milano Piazza Leonardo da Vinci 32 Milano 20133 Italy; ^2^ Department of Materials Science Università degli Studi di Milano‐Bicocca Via Roberto Cozzi 55 Milano 20125 Italy; ^3^ Department of Chemistry University of Torino Via Pietro Giuria 7 Torino 10125 Italy; ^4^ Nanostructured Interfaces and Surfaces (NIS) Interdepartmental Centre University of Torino Via Pietro Giuria 7 Torino 10125 Italy; ^5^ Present address: School of Water and Environment Key Laboratory of Subsurface Hydrology and Ecological Effect in Arid Region of the Ministry of Education Chang'an University Xi'an 710064 China

**Keywords:** catalyst structure, DFT calculations, single‐atom catalysis, solid‐state chemistry, X‐ray absorption spectroscopy

## Abstract

Single‐atom catalysts (SACs) are gathering significant attention in chemistry due to their unique properties, offering uniform active site distribution and enhanced selectivity. However, their precise structure often remains unclear, with multiple models proposed in the literature. Understanding the coordination environment of the active site at the atomic level is crucial for explaining catalytic activity. Here, a comprehensive study of SACs made of carbon nitride (CN_x_) containing isolated nickel atoms is presented. Using a combination of synthesis techniques and characterization methods including Fourier‐transform infrared spectroscopy, X‐ray absorption spectroscopy (XAS), and density functional theory (DFT) calculations, the local environment of nickel active centers in CN_*x*_‐supported SACs is investigated. These results challenge conventional structural models and propose a new architecture that better aligns with current experimental evidence. This new structure serves as a foundational step toward a rational approach to catalyst development and can facilitate more precise design and application of these innovative catalysts.

## Graphical abstract

This study investigates the nanostructure of Ni‐based single‐atom catalysts supported on carbon nitride. By combining synthesis, advanced characterizations, and DFT calculations, the study reveals a new architecture for the nickel active sites, challenging previous models. These findings offer a foundation for more precise design and application of single‐atom catalysts.

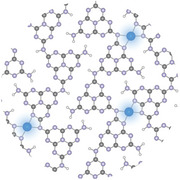

## 1 Introduction

The quest to understand the structure of a catalyst is pivotal in chemistry and materials science.^[^ ^1^ ^]^ This understanding serves as a cornerstone for the rational development of catalysts and can significantly enhance the efficiency and specificity of catalytic processes. In this context, single‐atom catalysts (SACs) have emerged as a frontier in catalysis research due to their unique set of properties.^[^ ^2^, ^3^ ^]^ The isolated nature of the active metal atoms in these catalysts offers a series of practical advantages, including uniform active site distribution, maximized metal utilization, and distinct electronic properties that can be finely tuned to enhance selectivity in a variety of applications ranging from energy conversion to organic synthesis and environmental remediation. Among the support materials available for SACs, carbon nitride (CN*_x_*) has been extensively adopted as a support for metal immobilization,^[^ ^4^, ^5^, ^6^, ^7^, ^8^, ^9^ ^]^ and this success is inherent to its versatile physicochemical properties, thermo‐ and photostability, tunable surface area, and synthesis routes from cheap and readily available precursors (e.g., cyanamide, melamine).^[^ ^10^, ^11^ ^]^ Substantial efforts have focused on developing novel synthesis protocols and exploring their advanced uses in model reactions and other complex applications, including selective hydrogenations, gas reduction reactions, cycloadditions, and energy‐related electrochemical processes, where they demonstrated superior performance compared to cluster‐ or nanoparticle‐based counterparts due to their remarkable activity, stability, selectivity, and reusability (**Table** **1**). Readers interested in the applications of SACs are referred to recent reviews where the broad and diverse use of these catalysts is comprehensively described.^[^ ^2^, ^12^ ^]^

**Table 1:** Key applications of Ni‐based SACs.

Catalytic application	Refs.
***Photocatalytic reactions***	
Hydrogen and H_2_O_2_ production	[13, 14, 15]
CO_2_ reduction	[16, 17]
CO_2_ methanation	[18]
Hydrogenation	[19]
Pollutant degradation	[20, 21]
C─N and C─O cross‐coupling reactions	[22, 23, 24]
***Electrocatalytic reactions***	
Hydrogen evolution reaction (HER)	[25, 26]
Oxygen evolution reaction (OER)	[27 ]
Oxygen reduction reaction (ORR)	[28, 29]
Carbon dioxide reduction reaction (CO_2_RR)	[30]
Nitrogen reduction reaction (NRR)	[31]
Electrochemical sensing	[32, 33]
Electrosynthesis of dimethyl carbonate and ammonia	[34, 35]

**Figure 1:**
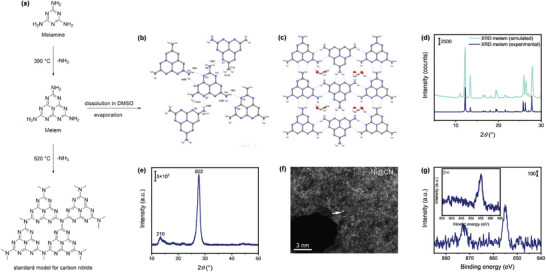
Characterization of melem and nickel single‐atom catalyst. (a) Sketch of the synthesis of carbon nitride from melamine. (b) Crystallographic representation (ellipsoid 50% probability) of the 2D layered structure in the hydrated melem formed by hydrogen bonding network and (c) of the 2D layered structure with the disordered water molecules. Atom color code: grey = carbon, blue = nitrogen, red = oxygen, and white = hydrogen. Hydrogen bonds are indicated as light blue lines. (d) Powder XRD diffraction analysis showing the pattern of the isolated melem powdered (blue line) and that simulated from the single crystal structure of VANXIS (green line) of hydrated melem. (e) XRD pattern of Ni_1_@CN*_x_* upon completion of the calcination step, and (f) HAADF‐STEM of the same sample corroborating the presence of Ni single atoms on the support. (g) High‐resolution Ni 2*p* XPS spectrum of Ni_1_@CN*_x_* with features at 855 and 872 eV assigned to the presence of Ni^2+^ species, with the corresponding Auger electron spectroscopy profile as an inset.

Despite the potential of SACs, a thorough understanding of their precise structure remains elusive. In particular, numerous structural models have been documented in scholarly literature. One of the most widely reported frameworks encompasses the presence of heptazine units (**Figure** **1**a).^[^ ^11^ ^]^ Slight variations of this model have been also reported, including ionic heptazine‐based CN*_x_* represented by potassium polyheptazine imide,^[^ ^36^, ^37^ ^]^ polytriazine imide with intercalated LiCl,^[^ ^38^ ^]^ and melon‐type architectures.^[^ ^39^ ^]^ Several models can be found in the literature to describe the local environment of the active metal species and how the isolated active metals are coordinated by the neighboring atoms in CN*_x_* and analogous derivative structures, but none of these models can fit the experimental features for SACs. This lack of standardization complicates the development of universal principles for their design and application, hindering the optimization and predictability of the catalytic performance. Furthermore, incorrect assumptions about the local site coordination inevitably lead to incorrect models, resulting in a loss of credibility of the simulations. In this work, we now present an extensive and detailed study of CN*_x_*‐supported SACs featuring isolated Ni atoms as active metal species, scrutinizing the local environment and electronic properties of the Ni centers and providing insights into the overall structure of these sites.

## 2 Results and Discussion

A Ni‐containing SAC (Ni_1_@CN*_x_*) and the corresponding metal‐free support were synthesized via hard‐template‐assisted condensation of cyanamide.^[^ ^40^ ^]^ For the SAC, during the synthesis procedure (Supporting Information for details), an inorganic nickel salt (NiCl_2_) was introduced as a source of the individually isolated active centers to facilitate the formation and stabilization of Ni single‐atom sites within the CN*_x_* framework. Figure 1a illustrates the primary intermediate species generated during the thermocondensation step which is an integral part of the preparation strategy for the synthesis of CN*_x_*‐supported Ni SAC. During this step, crystals of melem are formed and could be isolated by interrupting the synthesis and allowing for slow evaporation at room temperature in a DMSO solution. Single crystal X‐ray studies revealed that the unit cell of the obtained crystals consisted of molecules of melem with water molecules (Figure 1b,c), the latter likely incorporated from ambient moisture during the slow evaporation process. Details related to crystallographic data and the structure refinement are summarized in the Supporting Information (Table S1, Supporting Information). The assembly of the melem chains was mainly driven by an extended network of hydrogen bonds occurring between the three NH_2_ groups and the nitrogen atoms of the *s*‐triazine core of adjacent melem units, with an average distance of 2.273 Å. These strong hydrogen bonds assembled the melem units in a 2D layered structure, which resembled the supramolecular analogue of the CN*_x_* network. The nitrogen atoms of the *s*‐triazine core not involved in the hydrogen bonding network acted as hydrogen‐bond acceptor sites for the water molecules. The individual layers interacted through π‐π interactions between the melem units, adopting an offset‐face‐to‐face parallel displaced interactions motif. Powder X‐ray diffraction (XRD) analysis of the obtained material indicated that the synthesized melem (Figure 1d) was pure and highly crystalline, with no additional phases, and its powder pattern matched with the simulated one of the anhydrous melem reported in the literature.^[^ ^41^ ^]^ Based on the analysis of this intermediate, it is apparent that the final material consists of condensed melem structures arranged in a more amorphous configuration. Table S2 (Supporting Information) details the elemental composition and the main textural properties of the samples upon completion of the calcination step. The materials exhibited C/N ratios ranging from 0.60 to 0.65, which are lower than the expected stoichiometric C/N ratio (0.75).^[^ ^42^ ^]^ This variability has been frequently observed in these materials and attributed to structural and chemical defects.^[^ ^42^ ^]^ However, even if defects are hypothesized, they are difficult to assess accurately. Additionally, the hydrogen content determined through elemental analysis suggested incomplete polymerization of the CN*_x_* support, possibly indicating alternative, less regular carbon nitride structures with disrupted polymerization patterns. Inductively coupled plasma optical emission spectroscopy (ICP‐OES) confirmed the successful introduction of Ni species in the CN*_x_* framework, resulting in a Ni loading of 0.48% on the Ni_1_@CN_*x*_ catalyst (Table S2, Supporting Information). The surface area of Ni_1_@CN*_x_* is 166 m^2^ g^−1^, comparable to that of pure CN*_x_*, indicating that the incorporation of nickel did not significantly alter the porous structure of the carbon nitride matrix or cause structural degradation. The N_2_ physisorption isotherms (Figure S1a, Supporting Information) clearly suggested the presence of a porous structure. Solid‐state NMR spectra for carbon nitride (Figure S1b, Supporting Information) revealed distinct chemical shifts corresponding to *sp^2^* hybridized carbons in aromatic rings and carbon atoms bonded to nitrogen. This confirmed the formation of the graphitic‐like structure typical of carbon nitride, with strong C─N bonding patterns consistent with the expected heptazine‐based units in the polymeric network. XRD measurements (Figure 1e) enabled to confirm the purity and crystallinity of the Ni SAC and metal‐free support, with peaks at 2θ = 13° and 27°, indicating in‐plane and interplanar structural packing motifs of the aromatic units typical of CN*_x_*‐based materials.^[^ ^43^, ^44^ ^]^ The absence of additional peaks in the diffractograms suggested a lack of major crystalline phases assigned to metal clusters or nanoparticles of considerable size. This result was confirmed by the evaluation of the Ni dispersion on the CN*_x_* support via high‐angle annular dark‐field scanning transmission electron microscopy (HAADF‐STEM) imaging.^[^ ^45^ ^]^ An example of HAADF‐STEM imaging of Ni_1_@CN*_x_* is illustrated in Figure 1f, confirming the individual and homogeneous dispersion of the active Ni sites across CN*_x_*. Furthermore, no regions of pronounced local intensity were observed in the Ni map, suggesting the absence of metal clusters or nanoparticles originating from Ni aggregation (Figure 1f). The high‐resolution Ni 2*p* XPS spectrum of Ni_1_@CN*_x_* (Figure 1g), exhibiting characteristic peaks at 855 eV and 872 eV, was indicative of Ni^2+^ species. This assignment was further corroborated by the corresponding Auger electron spectroscopy profile, which is included as an inset. These results conclusively demonstrated the presence of isolated Ni species uniformly distributed within the CN*_x_* framework. Additional characterization data for the Ni_1_@CN*_x_* and metal‐free catalysts – including time‐of‐flight secondary ion mass spectroscopy (ToF‐SIMS) – are provided in the Supporting Information (Tables S3,S4 and relative discussion) and are consistent with the general characteristics of carbon nitride‐based SACs.

Fourier‐transform infrared (FT‐IR) spectroscopy was employed to investigate the nickel sites in the catalysts. We selected carbon monoxide (CO) as a versatile probe molecule, sensitive to the oxidation state and location of metal sites, to gain insights into the surface properties of our catalysts.^[^ ^46^ ^]^ As shown in Figure S2 (Supporting Information), the infrared spectrum of the metal‐free CN*_x_* support displayed several important absorption features. In particular, the prominent peak at 810 cm^−1^ could be ascribed to the breathing mode of the aromatic units constituting the CN*_x_* architecture,^[^ ^47^ ^]^ and the signals in the 1750—1100 cm^−1^ region to the in‐plane stretching modes of the aromatic building blocks in CN*_x_*.^[^ ^48^ ^]^ The broad band between 3500 and 3000 cm^−1^ is due to primary or secondary amine groups in the support structure, and underscored once again the presence of structural defects in the carbon nitride.^[^ ^49^ ^]^ This frequency range partially overlaps with the *υ*(OH) stretching signal associated to the presence of water molecules adsorbed on the support although these were removed upon outgassing at 150 °C in vacuum conditions (Figure S2, Supporting Information). Finally, we observed a sharp signal at ≈2200 cm^−1^, ascribed to nitrile groups probably localized at the CN*_x_* edges, whose presence has already been documented in previous studies.^[^ ^50^, ^51^ ^]^ This peak provided additional evidence supporting the hypothesis of incomplete polymerization in CN*_x_*‐type materials.

**Figure 2:**
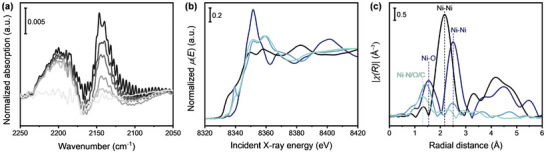
Analysis of Ni speciation in Ni_1_@CN*_x_* catalyst. (a) FT‐IR spectra of CO adsorption at 100 K at progressively decreasing partial pressures (from black to light grey) on the Ni_1_@CN*_x_* catalyst outgassed at 150 °C for 1 h; the background spectrum of the sample before CO adsorption was subtracted from all spectra. (b) Normalized Ni *K* edge XANES spectra, and (c) phase‐uncorrected FT‐EXAFS spectra of Ni_1_@CN*_x_*, Ni(II) phthalocyanine and Ni‐containing reference materials. Color codes: black = Ni foil, dark blue = NiO, blue = Ni(II) phthalocyanine, and light blue = Ni_1_@CN*_x_*.

As reported in **Figure** **2**a, upon CO dosage on the Ni_1_@CN*_x_* sample at cryogenic temperatures, the appearance of two main signals at ≈2200 and 2140 cm^−1^ was detected. Concerning the latter, its position close to gas phase CO (whose exact stretching mode appears at 2143 cm^−1^) and easy reversibility upon lowering the CO pressure suggested its assignment to weakly perturbed physisorbed CO.^[^ ^52^ ^]^ Conversely, the blue shifted band at ≈2200 cm^−1^ was attributed to Ni‐CO interactions, in agreement with previous spectroscopic investigations which reported a similar positive shift (i.e., +60 cm^−1^) for exposed Ni^2+^ ions dispersed on TiO_2_.^[^ ^53^ ^]^ Parallel FT‐IR investigations of the metal‐free CN*_x_* support (Figure S3, Supporting Information) resulted in spectra characterized by only weak signals, and dominated by gas phase and weakly adsorbed CO, proving that in the absence of Ni single atoms the interaction between CO and the CN*_x_* support was minimal and did not significantly contribute to the overall FT‐IR spectrum. The assignment of the Ni─CO band was supported by the analysis of a commercially available Ni(II) phthalocyanine, which possesses four nitrogen atoms available for metal coordination with the N species in a square‐planar geometry similar to that of Ni_1_@CN*_x_* catalyst (Figure 2b,c). In the spectra reported in Figure S4 (Supporting Information), we observed again the presence of a sharp peak at 2203 cm^−1^, ascribed to the interaction of the CO gas probe with the Ni^2+^ species bounded within the phthalocyanine cavity. The signal, in particular, was weak in intensity because the CO binding energy was expected to be very low for phthalocyanine moieties (see also DFT data).

XAS measurements were performed at the Ni *K* edge to investigate the electronic properties and local coordination environment of the active metal species in the Ni‐containing SAC, and to confirm the absence of clusters or nanoparticles resulting from metal atom aggregation. In Figure 2b, the X‐ray absorption near‐edge spectroscopy (XANES) spectra of the Ni catalyst are presented, alongside those of the reference materials such as nickel oxide (NiO), nickel metal foil (Ni foil), and Ni(II) phthalocyanine. XANES spectra indicated that in Ni_1_@CN*_x_*, the Ni atom adopts a square‐planar configuration, as evidenced by the specific energy absorption features characteristic of this geometry. The XANES pre‐edge region revealed dipole‐forbidden electronic transitions from 1*s* to 3*d* orbitals at ≈8333 eV, which were observable for all samples except for the Ni foil and are indicative of 3*d* orbital occupation and coordination geometry.^[^ ^54^ ^]^ The presence of a single quadrupole‐allowed 1*s*‐to‐3*d* transition peak in the pre‐edge region suggested $3d_{x^{2}-y^{2}}$ being the only empty 3*d* orbital, supporting the hypothesis of a square‐planar coordination geometry for the Ni species dispersed on the CN*_x_*‐supported SAC.^[^ ^54^ ^]^ Since 4*p* orbitals are generally vacant for Ni complexes, variations in energies related to electronic transitions to these orbitals, as well as to all high‐energy bound states, are affected by perturbations to the 4*p* energies and changes in the energy of the core orbitals due to variations in oxidation states.^[^ ^54^ ^]^ Features at the main absorption edge between 8345 and 8355 eV were attributed to dipole‐allowed electronic transitions from 1*s* to empty 4*p_x,y_* orbitals.^[^ ^34^ ^]^ The presence of additional spectral features, attributed to 1*s*‐to‐4*p_z_* transitions and localized between the pre‐edge and main edge both in Ni SAC and Ni(II) phthalocyanine supported the hypothesis of a planar coordinative architecture of the Ni sites (Ni‐N4) in the catalyst, without bonds along the 4*p_z_* orbital.^[^ ^30^, ^55^ ^]^ Figure 2c displays the Fourier‐transform extended X‐ray absorption fine structure (FT‐EXAFS) spectra of the Ni_1_@CN*_x_* catalyst together with Ni‐based reference materials. The corresponding imaginary parts of the FT‐EXAFS as well as the raw *k^2^*‐weighted *χ*(*k*) EXAFS spectra can be found in the Supporting Information. The absence of scattering contributions arising from metal–metal atomic pairs, evident in metal foils, pointed out to the presence of Ni species dispersed as individual atoms in our Ni catalyst.^[^ ^30^, ^55^ ^]^ Prominent peaks at 1.30–1.50 Å in the phase‐uncorrected FT‐EXAFS spectra could be assigned to Ni‐N first shell scattering, corroborating the coordination of Ni species to nitrogen atoms within SACs active centers. In fact, even if EXAFS alone cannot univocally determine the specific chemical elements coordinating isolated metal atoms in a catalytic system, the similarity of the FT‐EXAFS spectra of the Ni SAC and Ni(II) phthalocyanine allowed us to infer that Ni species are coordinated primarily by nitrogen atoms within the active sites of the catalyst. These findings consistently supported the single‐atom nature of the Ni SAC, the presence of Ni species in an average +2 oxidation state, and the architecture of their local environment characterized by the presence of nitrogen atoms coordinating the active metal species in a square‐planar geometry.

**Figure 3:**
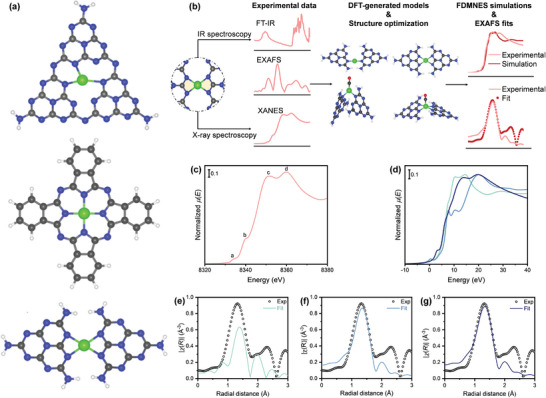
Data‐driven fitting of Ni single‐atom catalysts with predictive and experimental XANES and EXAFS spectra. (a) DFT‐generated model structure of a metal atom in the heptazine pore of C_3_N_4_ (Ni@HP‐CN*_x_*, top), in a square‐planar geometry (Ni@4N‐Pc, middle), and proposed carbon nitride structure (Ni@HL‐CN*_x_*, bottom). Extended structures are included in the Supporting Information. Color code: dark grey = carbon, blue = nitrogen, green = Ni atoms. (b) Workflow of the combined theoretical‐experimental approach where DFT‐based structures were fitted with the XAS spectra (other fittings are reported in the Supporting Information). (c) Experimental Ni *K* edge XANES spectrum of Ni_1_@CN*_x_*. (d) FDMNES simulations with the proposed DFT structures (Ni@HP‐CN*_x_*, Ni@4N‐Pc, Ni@HL‐CN*_x_*) related to the Ni SAC at *R* = 6 Å. The bottom panels present the phase‐uncorrected FT‐EXAFS spectra, *k^2^*‐weighted Fourier‐transformed EXAFS data (*χ*(*R*)) plotted as a function of radial distance (*R*), showing the first‐shell EXAFS fitting performed with the corresponding DFT structures: (e) Ni@HP‐CN*_x_*, (f) Ni@4N‐Pc, Ni@HL‐CN*_x_*.

The experimental results discussed above challenge the conventional model of CN*_x_* architecture, typically portraying heptazine cavities coordinating the active metal species in a distorted manner with four nitrogen atoms (**Figure** **3**a, top).^[^ ^4^, ^5^, ^6^, ^7^, ^8^, ^9^, ^56^ ^]^ This model has been widely used for simulating the stability and reactivity of SACs in several reactions.^[^ ^56^, ^57^ ^]^ However, some aspects of this model are inconsistent with spectroscopic evidence from XAS measurements and other experimental considerations reported above. First, a Ni atom in the traditionally reported heptazine pore of C_3_N_4_ (Figure 3a, top) cannot assume a configuration with four equivalent or nearly equivalent Ni‐N distances, as it has been widely reported in EXAFS data found in the literature and corroborated by the spectroscopic measurements presented in this study. The only way to reconcile the C_3_N_4_ model with experimental data is to assume that at room temperature the Ni atoms display a dynamic behavior within the heptazine pore, so that the interatomic distances obtained from the EXAFS spectra would represent an average of multiple bonding configurations. This, however, would raise questions regarding the stability of SACs in terms of diffusion and aggregation in thermal catalysis, as well as their dissolution or precipitation as oxide or hydroxide in electrocatalysis. While this aspect has been reported,^[^ ^58^, ^59^, ^60^ ^]^ it is generally valid for oxide supports, while CN*_x_*‐based SACs show enhanced stability at high temperatures (300 °C and above).^[^ ^61^ ^]^

An alternative model, also proposed in the literature for carbon‐based support materials,^[^ ^62^ ^]^ envisions a Ni atom surrounded by four N atoms in a square‐planar coordination within a di‐carbon vacancy of N‐doped graphene. However, this would imply a total restructuring of the molecular precursors used for the synthesis of the CN*_x_* matrix with the formation of a local structure resembling that of N‐doped graphene and is not consistent with the isolation of melem as an intermediate during materials calcination (Figure 1a–d). Nevertheless, in our DFT simulations, we have considered both models – Ni trapped in the heptazine pore of C_3_N_4_ (Ni@HP‐CN*_x_*) and Ni stabilized in an N‐doped graphene structure (Ni@4N‐Gr), to examine the properties of an adsorbed CO probe molecule on the Ni sites. The first system was simulated by means of a periodic model. Here, two elements were in contrast with the experimental evidence: beside the already mentioned asymmetric position assumed by the Ni atom (Figure 3a, top), a net red shift of the CO vibrational frequency was found (−75 cm^−1^, see Figure S5, Table S5, Supporting Information), while the FT‐IR data showed a positive blue shift of ≈+60 cm^−1^ (Figure 2a). A similar positive shift, +60 cm^−1^, has already been reported in previous works for exposed Ni^2+^ ions dispersed on TiO_2_.^[^ ^53^ ^]^ Therefore, we ruled out the Ni@HP‐CN*_x_* structures and started to investigate the four‐coordinated Ni atom using molecular models. The simulation of charged systems is commonly performed using molecular models,^[^ ^63^, ^64^, ^65^ ^]^ as this approach allows for flexible adjustment of the charge state without the complications related to the replication of charged species that may arise in periodic calculations. The reliability of this strategy was evaluated by comparing it to experimental benchmark data available in the literature, as detailed below.

To describe Ni@4N‐Gr, we employed a molecular analogue consisting of a Ni atom stabilized in a phthalocyanine molecule (Ni@4N‐Pc, Figure 3a, middle). Figure S6 (Supporting Information) compares the structure of the periodic Ni@4N‐Gr system and the molecular analogue, Ni@4N‐Pc, and it is possible to appreciate that the local coordination of the single‐atom is identical. This approach enabled us to explore different charge states of the Ni ion. The efficacy and reliability of this method and related models were validated against some simple [Ni*^y^* CO]*^x^* molecular complexes, where *x* is the total charge of the complex (i.e., *x* = 0, +1, and +2), demonstrating excellent reproduction of the observed CO vibrational frequencies in the corresponding FT‐IR spectra (Table S6, Supporting Information). Three charged states of the [Ni@4N‐Pc] complex were considered, namely 0, +1, and +2 (Table S7, Supporting Information). The CO molecule exhibited very weak binding via dispersion interactions to the [Ni@4N‐Pc] complex, irrespective of the net charge, as indicated by the adsorption energy (Δ*E*) values ranging from −0.12 and −0.17 eV. The distances between CO and the Ni site in the complexes (*r*(Ni‐C)) were quite long (≈3.3 Å), and the positive shifts in CO vibrational frequencies observed for the charged complexes were entirely attributed to the electric field generated by the positive charge. The highest computed CO binding energy, −0.17 eV, corresponded to a desorption temperature of ≈50 K, below the temperature at which the FT‐IR experiments were conducted. Therefore, the Ni@4N‐Pc model was discarded based on three considerations: i) it is highly improbable that this structure forms during the synthetic procedure; ii) the structure can only bind CO at extremely low temperatures; iii) even for a complex in a +2 charged state, the CO vibrational shift (+27 cm^−1^) is well below that measured experimentally.

Clearly, the structural models typically reported in the literature to describe SACs based on CN*_x_* do not align with experimental data. To address this discrepancy, we explored alternative structures and hypothesized that Ni atoms or ions in CN*_x_*‐supported SACs form complexes with heptazine molecules or polymeric structures during catalyst synthesis. This hypothesis is supported by the formation of the melem intermediate – which has been isolated and characterized (Figure 1a–d) – during the condensation of the CN*_x_* precursors (e.g., cyanamide or melamine).^[^ ^41^ ^]^ This structure can coordinate metal species via the nitrogen atoms belonging to the heptazine ligands. Based on this, we constructed, among other hypotheses, a Ni@HL‐CN*_x_* complex where Ni is directly coordinated to the N atoms of the heptazine units (Figure 3a, bottom), and we considered three charged states – 0, +1, and +2 (Figures S7–S9, Table S8, Supporting Information, and relative discussion). Importantly, the Ni atoms always assumed a Ni(II) formal oxidation state, in agreement with the experimental value. The corresponding complexes were fully optimized, and their interaction with CO were studied using Gaussian09 calculations. The target properties we aimed to reproduce included the coordination of Ni species within an approximately square‐planar coordination. Additionally, it was essential for the CO to bind to the metal center with a binding energy of at least 0.5 eV, ensuring stability at ≈100 K. Furthermore, we sought to observe a blue shift in the C─O stretching frequency in the FT‐IR spectra of ≈+60 cm^−1^ compared to the gas‐phase value of 2143 cm^−1^. For the doubly charged [Ni@HL‐CN*_x_*]^2+^ complex, consistent with the experimentally observed Ni^2+^ valence state, we identified two electronic states – singlet (S) and triplet (T) – very close in energy (the triplet is 130 meV more stable than the singlet, within the accuracy of DFT calculation of ≈0.1 eV). The structure of the [Ni@HL‐CN*_x_*]^2+^ complex remained consistent in both cases, with Ni bound in a slightly distorted square‐planar configuration to four N atoms (Figures S7c,S8f, Supporting Information). As shown in Table S8 (Supporting Information), in the singlet state of the complex, the Ni─N bond distances are 1.92–1.95 Å, while in the triplet state, they are ≈0.1 Å longer (2.00–2.04 Å). In both states of the complex, CO is strongly bound to the Ni site, resulting in a significant blue shift in the CO stretching frequency (+42 cm^−1^ for the singlet, +77 cm^−1^ for the triplet). Given that the experimental shift in the FT‐IR spectra of the Ni SAC was ≈+60 cm^−1^, we concluded that both complexes showed a shift compatible with that measured experimentally. It is essential to note that the presence of a Ni^2+^ ion bound to the support material is fundamental for reconciling theoretical calculations with experimental measurements. A similar observation was reported in the discussion concerning vibrational spectra of CO adsorbed on exposed Ni^2+^ ions on silica.^[^ ^66^ ^]^

*Ab initio* finite difference method for near‐edge structure (FDMNES) simulations and high‐throughput EXAFS fitting of the experimental XAS spectra (Figure 3b) further validated the local coordination environment of active metal centers in Ni_1_@CN*_x_* employing the DFT‐based structures described above (Ni@HP‐CN*_x_*, Ni@4N‐Pc, Ni@HL‐CN*_x_*). The XANES and EXAFS regions of XAS spectra were analyzed to extract distinct electronic and structural information concerning the Ni SAC. Therefore, a combined fitting on both these regions was performed for the first time on a single‐atom catalyst, elucidating spectral features arising from subtle structural variations. The experimental Ni *K* edge XANES spectrum and the corresponding FDMNES simulations for the three DFT‐based structures are shown in Figure 3c,d, respectively. The experimental Ni *K* edge XANES spectrum of the catalyst displays four distinct features, including a doublet (Figure 3c, “c”: 8351 eV and “d”: 8260 eV) at the main edge and two small shoulders (Figure 3d, “a”: 8334 eV and “b”: 8340 eV) at the rising edge. In the FDMNES simulations, all atoms within a radius of 6 Å centered on the absorbing Ni metal ion were considered. Consistent convolution parameters were used to convolute the three calculated spectra and are provided in Table S9 (Supporting Information). The calculated XANES spectra accurately reproduced the characteristic features of the experimental Ni single‐atom spectrum and their relative intensities. The main doublet feature observed in the latter on the absorption edge is well replicated by both the conventional Ni@HP‐CN*_x_* structure and the newly proposed Ni@HL‐CN*_x_* model. However, “c” and “d” features were better resolved in terms of their relative intensity and energy separation when considering the Ni@HL‐CN*_x_* model. Additionally, the shoulder features “a” and “b” observed at the rising edge of the experimental XAS spectrum of the Ni SAC were also reproduced by the novel model structure. In contrast, the Ni@4N‐Pc XANES spectrum exhibited unique signatures at both the rising and absorption edges that were not present in the experimental XAS output. The Ni@4N‐Pc XANES region differs significantly in terms of spectral profile and relative intensities of characteristic features in comparison to the simulated Ni@HP‐CN*_x_*, Ni@HL‐CN*_x_*, and experimental spectra. The results of XANES calculations using DFT‐based structural models indicated that, among the three simulated XANES spectra, the newly proposed Ni@HL‐CN*_x_* structure displays the closest agreement with the experimental spectroscopic measurements.

**Figure 4:**
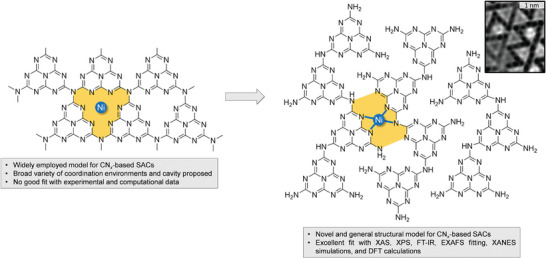
Structural understanding of carbon nitride‐based SACs. Spectroscopic evidence confirms that Ni@HL‐CN*_x_* serves as a precise model for SACs, revealing that during polymerization, melem units join into polymeric CN*_x_* structures by pairing up, forming filaments that trap metal atoms. This refined structural hypothesis is further supported by recent atomic force microscopy studies on metal‐free carbon nitride layers,^[^ ^67^ ^]^ and a representative micrograph is shown as an inset in Figure 4.

First‐shell EXAFS fitting of the experimental Ni *K* edge spectra of the Ni‐containing SAC was performed in the 1–2.3 Å Δ*R* range. Working in *R* space allowed us to selectively ignore higher coordination shells. A preliminary first‐shell EXAFS fitting of the Ni(II) phthalocyanine model compound (Table S10, Figure S13, Supporting Information) was also performed to determine the *S_0_^2^* parameter. Figure 3e–g shows the phase‐uncorrected FT‐EXAFS spectra as a function of radial distance for the three DFT structures (Ni@HP‐CN*_x_*, Ni@4N‐Pc, Ni@HL‐CN*_x_*) used as starting points to build the EXAFS fitting model. To effectively differentiate the structural variations in the proposed DFT structures, a systematic approach was adopted. Hence, EXAFS fitting was exclusively performed for the first shell, including contributions from the nearest neighbors to the Ni atom. The selected Δ*R* range ensured the consideration of Ni‐N contributions only, eliminating Ni‐C scattering paths, and was consistently applied to all three structural models. This strategy minimized the number of EXAFS fitting parameters, ensuring a robust fit in all cases. To model the first‐shell EXAFS spectra, we used the computed EXAFS signal stemming from the Ni‐N scattering paths and refined the values for *E_0_*, *σ^2^*, and Δ*R*. For the structural refinement, the amplitude (*S_0_^2^*) of the FT‐EXAFS signal was kept fixed during the fitting. All the parameters related to the EXAFS fitting procedures and corresponding error bars are provided in Table S11 (Supporting Information). In the first shell of the Ni@HP‐CN*_x_* model, Ni is coordinated by three nitrogen atoms with an average Ni─N bond distance of ≈2.1 Å. A major key observation related to the EXAFS fitting of the Ni@HP‐CN*_x_* structure was the amplitude of the first‐shell feature (Figure 3e), where the reduced intensity of this peak may derive from the interference of anti‐phase back‐scattering waves due to the pronounced asymmetry in the Ni–N interatomic distance distribution predicted by DFT. The low quality of the fit is reflected in the high value of the *R* factor and excessively small value of *σ^2^* below 0.001 Å^2^. On the other hand, the fits for both Ni@HL‐CN*_x_* and Ni@4N‐Pc models were found to be in closer agreement with the experimental EXAFS spectrum, showing low values of *R* factor and more reasonable values of *σ^2^* ≈ 0.006–0.008 Å^2^ (Figure 3f,g), denoting a relatively high level of local structural disorder in the first‐shell distance distribution. The intensities of the feature related to the first shell in the Ni@HL‐CN*_x_* and Ni@4N‐Pc spectra were also well reproduced (Figures S10–S12, Supporting Information). Overall, both Ni@4N‐Pc and Ni@HL‐CN*_x_* possess four nitrogen atoms in the first shell with an average Ni─N bond distance of ≈1.9–2.0 Å, indicative of strong metal–nitrogen interactions. However, Ni@4N‐Pc exhibits several structural and electronic properties that are in contrast with the evidence arising from the characterization of the synthesis intermediates, the FTIR spectra, and the DFT modelling, including the different CO binding properties. In addition, it results into a worse reproduction of the experimental curve, when used to simulate the Ni *K* edge XANES spectrum. It is thus the combination of these techniques that collectively indicated that the proposed Ni@HL‐CN_*x*_ structure is the only configuration consistent with the observed experimental data. The consistency across these spectroscopic techniques not only confirms that Ni@HL‐CN*_x_* is an accurate and reliable model for metal‐based carbon nitride SACs but also alters our interpretation of the structure of SACs. The findings align with the collected spectroscopy and support the hypothesis that during polymerization, melem units assemble into a polymeric CN*_x_* where only two melem units are joined together, forming filaments where the metal is then entrapped. This hypothesis is further validated by recent atomic force microscopy (AFM) studies on carbon nitride layers (**Figure** **4**), which show a strong correlation with the proposed molecular model for CN*_x_*‐based SACs. The AFM image displays a consistent arrangement, homogeneous and well‐defined, that is attributed by the heptazine units forming the core of the support material. This pattern emerging from the AFM image aligns with the novel structural model Ni@HL‐CN*_x_*, where Ni atoms are coordinated by nitrogen in a square‐planar configuration. Moreover, the 1 nm scale bar confirms that the features observed in the AFM image correspond to atomic‐level details, further supporting the conclusion that the image provides direct physical evidence for the stability and structure of the proposed CN*_x_*‐based SAC model.

Even though we acknowledge that the new structural model presented herein may not universally apply across the whole SAC field, we are confident in the general relevance of our findings for carbon CN*_x_*‐supported SACs. We employed EXAFS spectroscopy to evaluate the effectiveness of traditional structural models in representing experimental data in comparison to our newly proposed architecture. Our analysis demonstrated that many established architectures of the CN*_x_* support fail to adequately capture the spectroscopic features observed in the experimental data, whereas the novel Ni@HL‐CN*_x_* structure consistently aligns with these results, suggesting its broader applicability. This can be rationalized by the observation that all synthetic protocols for this catalyst family converge on “melem” intermediates – a critical transition step we propose as essential for stabilizing single‐atom configurations in CN*_x_*‐based SACs. Supported by spectroscopic data and computational simulations, we are confident that the new structural model proposed here represents a broadly applicable framework for SACs supported on CN*_x_*.

## 3 Conclusion

This work represents the first detailed investigation of the newly proposed structure for carbon nitride‐based single‐atom catalysts, and it is sustained by a robust integration of spectroscopic techniques, analytical methods, and a wide set of density functional theory simulations. In fact, through a comprehensive investigation utilizing synthesis methods, spectroscopic techniques, and density functional theory simulations, we have elucidated the electronic nature and the local coordination environment of nickel active centers in CN*_x_*‐supported SACs. The convergence of results from these diverse methodologies strongly substantiates our findings and challenges existing structural models, leading us to propose a new architecture that more accurately accounts for experimental observations. The identified structural unit in CN*_x_*, characterized by a tetracoordinated Ni^2+^ cation with average Ni─N bond distances of ≈1.95 Å, simultaneously ensures the best reproduction of the experimental features in XANES and EXAFS spectra, incorporating all characteristics sensitive to both regions. The results are also corroborated by FT‐IR spectroscopic analyses with probe molecules and by the observation of a charged Ni^2+^ state. The identification of tetracoordinated Ni^2+^ cations in the “resting” state of Ni SACs is a crucial first step toward the understanding of a catalytic mechanism, even when the oxidation state of the isolated metal may change based on the specific reaction conditions during a catalytic cycle. Overall, this work contributes to the advancement of SAC research and provides new insights for the rational design and optimization of catalysts with enhanced catalytic performance.

## 4 Experimental Section

### Catalyst Preparation

Nickel(II) chloride (21 mg; Sigma‐Aldrich, 98%) and cyanamide (3 g; Sigma‐Aldrich, 99%) were added to an aqueous suspension of silica (7.5 g; Sigma‐Aldrich, SiO_2_ Ludox HS‐40) serving as a hard template for the formation of mesopores in the support material. The mixture was stirred and heated at 70 °C for 16 h to facilitate water evaporation, and the resulting white solid was calcined at 550 °C for 4 h using a temperature ramp of 2.2 °C min^−1^. The as‐obtained material was added to a 4.2 m solution of ammonium bifluoride (Sigma‐Aldrich, 95%), and then kept at room temperature for 48 h while stirring to remove the silica template. The dispersion was subjected to a centrifugation step (5000 rpm, 5 min), and the precipitate was washed with water and ethanol. Last, the catalyst was dried at 70 °C overnight under vacuum conditions to obtain the final CN*_x_*‐supported Ni single atoms. The metal‐free mesoporous graphitic CN*_x_* support was synthesized following the same preparation strategy, but without adding the nickel salt as a source of active metal species.

### Catalyst Characterization—Chemical analysis

The nickel content in the catalyst was measured through ICP‐OES using a Perkin Elmer Optima 8300 equipped with a photomultiplier tube detector. Upon the dissolution of the catalyst in a strong acidic medium and its nebulization to produce a fine aerosol, an inductively coupled plasma torch was employed to generate excited atoms and ions emitting photons at wavelengths characteristic of specific chemical elements, thus enabling the identification and quantification of the nickel loading on the catalyst. The C, N, and H composition of the samples was determined by a traditional combustion analysis carried out on a Vario MICRO Elemental Analyzer. The materials were subjected to high‐temperature treatment (>1000 °C), resulting in the combustion of the samples. The as‐produced gaseous molecules were subsequently separated by gas chromatography and quantified using a thermal conductivity detector.

### Low Temperature N_2_ Physisorption

Nitrogen physisorption measurements were conducted using a 3P Sync 400 instrument at −196 °C to evaluate the porous nature and surface areas of the samples. An outgassing step was carried out at 150 °C for 24 h before the textural measurements to remove any residual moisture or adsorbed contaminant from the catalytic surfaces. The specific surface areas were determined resorting to the Brunauer–Emmett–Teller method on the adsorption branch of the isotherms within the pressure range of 0.05 < *p*/*p_0_* < 0.3 as this corresponded to the transition from the monolayer to the multilayer of nitrogen gas molecules adsorbed on the catalyst surfaces.

### X‐Ray Diffraction (XRD)

XRD patterns were collected on a Bruker D2 Phaser X‐ray diffractometer equipped with a Cu *Kα* radiation source (*λ* = 0.15405 nm) within the 5–60° 2*θ* range. The XRD diffractograms were acquired with a 2*θ* step size of 0.016° and a counting time of 0.4 s per step. The samples, in powder form, were placed on flat alumina holders and directly analyzed in air without any further treatment.

### High‐Angle Annular Dark‐Field Scanning Transmission Electron Microscopy (HAADF‐STEM) Imaging

HAADF‐STEM analyses were carried out using an UltraSTEM 100 (Nion Co.) microscope equipped with a cold field emission electron source and a quadrupole–octupole aberration corrector in the probe‐forming electron optics. Given that carbon nitrides are prone to radiolytic damage under electron beam exposure,^[^ ^68^ ^]^ damage deriving from collisions with the incident electron beam was expected to be minimized at lower beam energies, motivating the use of 60 keV accelerating voltage. The convergence semi‐angle was fixed at 31 mrad, and the HAADF detector collection semi‐angles ranged from 90 to 190 mrad.

### X‐Ray Photoelectron Spectroscopy (XPS)

Surface investigations based on high‐resolution XPS were performed on the PHI 5000 Versa Probe II XPS system (Physical Electronics) with a monochromatic Al *Kα* source (15 kV, 50 W) and photon energy of 1486.7 eV. Dual‐beam charge compensation was used for all measurements. All spectra were measured in a vacuum (1.3 × 10^−7^ Pa). All binding energy (BE) values were referenced to the carbon peak C 1*s* at 284.80 eV.

### Time‐of‐Flight Secondary Ion Mass Spectrometry (ToF‐SIMS)

ToF‐SIMS analyses were performed with an ION‐TOF TOF.SIMS 5 reflectron‐based mass spectrometer equipped with a Bi‐cluster ion gun operated at 50 kV and a time‐of‐flight mass analyzer. Bi_3_^+^ primary cluster‐ions were selected. Charge neutralization was applied throughout the measurements employing a low energy electron flood source. For each sample, a layer of powder ≈0.5 mm thick was placed onto a sample holder comprising a silicon wafer covered with double‐sided sticky tape and flattened as much as possible using a spatula. The ToF‐SIMS analyses were conducted by recording the secondary ion mass spectra in the 1–876 atomic mass unit range, using a probing area of 100 × 100 µm^2^ on positions where the catalytic powders were completely covering the underlying carbon tape. The intensity of peaks in ToF‐SIMS plots was normalized to the total ion current. Data were analyzed using the SurfaceLab 7 software and libraries available from IONTOF.

### Fourier‐Transform Infrared (FT‐IR) Spectroscopy

FT‐IR measurements were performed employing a Bruker Equinox 55 spectrophotometer equipped with a Globar source and with a Mercury Cadmium Telluride (MCT) detector. For each sample, 128 interferograms acquired at a 4 cm^−1^ resolution were averaged in the 4000–400 cm^−1^ wavenumber range. Prior to conducting FT‐IR measurements, the samples in powder form were pressed to obtain a thin, self‐supporting pellet (with “optical density” < 10 mg cm^−2^) and then introduced into quartz cells with KBr windows allowing measurements at low temperature (≈100 K) by cooling with liquid N_2_.^[^ ^69^ ^]^ The cell was connected to a conventional vacuum line (at a residual pressure < 5.0 × 10^−4^ mbar), enabling all thermal treatments and adsorption–desorption experiments to be carried out in situ. Before performing CO adsorption measurements, the samples were heated under vacuum at 150 °C for 1 h to remove adsorbed water and surface contaminants. All FT‐IR spectra were normalized to the optical density of the pellets in order to facilitate quantitative comparisons.

### X‐Ray Absorption Spectroscopy (XAS)

Ni *K* edge XAS spectra were collected at the BM23 beamline of the European Synchrotron Radiation Facility (ESRF) in Grenoble, France.^[^ ^70^ ^]^ The end station of the BM23 beamline was equipped with a water‐cooled double‐crystal monochromator (flat Si[111] pair) to scan the incident photon energy and the two ionization chambers (filled with He/Ar mixture) to detect the incident (*I_0_*) and transmitted (*I_1_*) X‐ray photons. A nickel metal foil, typically used as a reference material, was measured simultaneously with all the acquired XAS spectra through a third ionization chamber (*I_2_*) to facilitate accurate edge energy calibration and spectra alignment in the subsequent data analysis.^[^ ^71^, ^72^ ^]^ The powdered samples – catalyst and the reference compounds – were prepared in the form of self‐supporting pellets with a 5‐mm diameter, and masses were optimized for XAS data collection in transmission mode at room temperature (optimized sample weight for Ni SAC is 74 mg, resulting in edge jump Δ*μx* = 0.51 for a total absorption after the edge of *μx* = 2.5). Despite the relatively low nickel content in the SAC, XAS spectra were acquired in transmission mode due to the light composition of the CN*_x_* matrix mainly comprising of carbon and nitrogen atoms. Each XAS spectrum was collected with an acquisition time of ≈3 min scan^−1^, and the energy of the X‐ray beam of the incident photons was scanned across the Ni *K* edge (8333 eV). All spectra were collected in continuous mode with an energy step of 0.3 eV. Background subtraction, energy alignment, and edge jump normalization were carried out with the ATHENA software as part of the Demeter suite.^[^ ^73^ ^]^ The final spectra presented in this study were obtained as the average of five spectra per sample after checking the reproducibility of the measurements among the scans. Following the extraction of the extended X‐ray absorption fine structure (EXAFS) functions in the *E* space (*χ*(*E*)) and the conversion of energy into wavenumbers to get *χ*(*k*) in the *k* space, the FT‐EXAFS spectra were obtained applying the Fourier transform on the *k^2^*‐weighted *χ*(*k*) functions in the 2.5–10 Å^−1^ *k* range.

### XANES Simulations

*Ab initio* simulations of the XANES regions of XAS spectra were performed using the FDMNES code developed by Joly et al.^[^ ^74^ ^]^ This code provided users with two main approaches for calculating XANES spectra: the finite difference method (FDM), which is a full potential approach, and the multiple scattering theory, that employ the so‐called muffin‐tin approximation on the potential shape in a fully relativistic frame. In the present study, FDM was selected to simulate near‐edge spectral features of the Ni *K* edge XAS by using self‐consistency in the evaluation of the final states. Simulations were carried out employing three DFT‐optimized structures of the Ni SAC considering all atoms within a radius of 6.0 Å centered around the absorbing Ni atom. In FDM, the energy‐dependent exchange‐correlation potential was obtained from the Hedin–Lundqvist approach using local density approximation which avoided the muffin‐tin approximation. Once this potential function was obtained, Schrodinger‐like equations were solved to get the electronic structures of the Ni centers of the investigated catalysts. Additional details regarding the XANES simulations – including the convolution parameters and convergence test – are provided in Table S9 and Figures S10,S11 (Supporting Information).

### EXAFS Fitting

EXAFS fitting for the Ni SAC sample was performed in *R* space, in the Δ*R* = 1.0–2.3 Å range, on the FT‐EXAFS spectrum obtained by applying the Fourier transform on the *k^2^*‐weighted *χ*(*k*) functions in the 2.5–10 Å^−1^ *k* range. Working in R space allowed us to selectively ignore higher coordination shells, performing only the first shell EXAFS fitting as a systematic approach to study structural variations in the three DFT‐optimized structures of the Ni SAC. Scattering amplitudes (f(*k*)) and phase‐shifts (*δ*(*k*)) were calculated by FEFF6 code using the Artemis software from the Demeter package.^[^ ^73^ ^]^ Following the calculation of the electron scattering paths by the cluster of atoms surrounding the Ni center in the Ni SAC, FEFF6 generated an output file detailing the scattering contributions to the EXAFS spectra within each scattering path. Detailed information related to the parameters employed and optimized in the EXAFS fitting is reported in Table S11 (Supporting Information).

### DFT Calculations—Computational Methods for Molecular Complexes

The calculations of CO infrared spectra with DFT approaches required the use of self‐interaction corrected functionals (e.g., hybrid functionals), as standard GGA functionals overestimated the metal‐to‐CO back‐donation and resulted in unphysical red shifts. It was relied on molecular calculations to account for the possible formation of charged systems. In fact, the properties of adsorbed CO could hardly be evaluated within periodic approaches due to problems related to charged supercells. Spin‐polarized (Unrestricted) DFT calculations were performed with the Gaussian 09 code,^[^ ^75^ ^]^ by using the PBE0+D3 hybrid functional.^[^ ^76^ ^]^ Optimization and frequency calculations were conducted utilizing the same basis set 6–31++G(d, p) with diffuse functions for all atoms.^[^ ^77^, ^78^ ^]^ Additionally, a 6–31G(d, p) basis set was employed for larger models to ensure smooth executions of the computations. Default convergence criteria were adopted on both maximum displacements and root mean square of energies and gradients.

### Computational Methods for Periodic Complexes

Spin‐polarized DFT calculations were performed with the VASP code,^[^ ^79^, ^80^, ^81^ ^]^ by considering the generalized gradient approximation as implemented in the Perdew–Burke–Ernzerhof (PBE) functional.^[^ ^82^ ^]^ Dispersion forces were taken into account according to Grimme's D3 parametrization.^[^ ^83^ ^]^ Valence electrons for each atom were expanded on a set of plane waves with a kinetic energy cutoff of 400 eV, whereas the core electrons were treated with the projector augmented wave approach and by adopting the recommended pseudopotential from VASP.^[^ ^84^, ^85^ ^]^ The threshold criteria for electronic and ionic loops were set to 10^−6^ eV and 10^−3 ^eV Å^−1^, respectively. Additionally, to more accurately describe the Ni properties, the Hubbard correction was applied by considering a *U*‐value equal to 3.40 eV, as reported in the original work of Solovyev and corroborated by previous research.^[^ ^86^, ^87^ ^]^

## Conflict of Interest

The authors declare no conflict of interest.

## Author Contributions

N.A., S.X., and S.F.J. contributed equally to this work. G.V. conceived and coordinated the work. NA prepared the samples. N.A. and G.T. conducted preliminary characterization studies (XRD, crystallographic studies, N_2_ physisorption, ICP‐OES, and CHNS analysis). N.A. and L.M. performed gas probe‐based FTIR spectroscopy measurements. N.A., S.F.J., L.M., and E.B. carried out EXAFS experiments and analyzed the data. G.V. proposed possible molecular structures based on single‐crystal experiments. G.P., G.D.L., S.T., S.X., and L.A.C. performed DFT calculations. All authors contributed to writing the present manuscript and gave their approval to the final version.

## Supporting information



Supporting Information

## Data Availability

The data that support the findings of this study are available from the corresponding author upon reasonable request.
