# Supplementary material for: Resolving the Nanostructure of Carbon Nitride‐Supported Single‐Atom Catalysts
Source: Small. 2025 Jan 9;21(23):2408286. doi: 10.1002/smll.202408286 (PMC12160693; doi:10.1002/smll.202408286)
Supplement: Supplementary file 1 — Supporting Information [file SMLL-21-2408286-s001.pdf]

NANO · MICRO
small

Supporting Information

for *Small*, DOI 10.1002/smll.202408286

Resolving the Nanostructure of Carbon Nitride-Supported Single-Atom Catalysts

Nicolò Allasia, Shuai Xu, Sadaf Fatima Jafri, Elisa Borfecchia, Luis A. Cipriano, Giancarlo Terraneo, Sergio Tosoni, Lorenzo Mino, Giovanni Di Liberto, Gianfranco Pacchioni* and Gianvito Vilé**

Supporting Information

Resolving the Nanostructure of Carbon Nitride-Supported Single-Atom Catalysts

Nicolò Allasia,[a,†] *Shuai Xu,*[b,†,§] *Sadaf Fatima Jafri,*[c,d,†] *Elisa Borfecchia,*[c,d] *Luis A. Cipriano,*[a] *Giancarlo Terraneo,*[a] *Sergio Tosoni,*[b] *Lorenzo Mino,**[c,d] *Giovanni Di Liberto,*[b] *Gianfranco Pacchioni,**[b] *and Gianvito Vilé**[a]

[a] *Department of Chemistry, Materials, and Chemical Engineering "Giulio Natta", Politecnico di Milano, Piazza Leonardo da Vinci 32, 20133 Milano, Italy.*

[b] *Department of Materials Science, Università degli Studi di Milano-Bicocca, Via Roberto Cozzi 55, 20125 Milano, Italy.*

[c] *Department of Chemistry, University of Torino, Via Pietro Giuria 7, 10125 Torino, Italy.*

[d] *Nanostructured Interfaces and Surfaces (NIS) Interdepartmental Centre, University of Torino, Via Pietro Giuria 7, 10125 Torino, Italy.*

† *Nicolò Allasia, Shuai Xu, and Sadaf Fatima Jafri contributed equally to this work.*

§ *On leave from: School of Water and Environment, Key Laboratory of Subsurface Hydrology and Ecological Effect in Arid Region of the Ministry of Education, Chang'an University, Xi'an 710064, China*

Supporting characterization results

Crystallographic studies on melem

Table S1. *Crystallographic data and details of the structure refinement for melem structure.*

Chemical formula	$C_6H_6N_{10}$, H_2O
Molecular weight (g mol^{-1})	236.22
Crystal system, space group	Monoclinic, $P2_1/c$
Temperature (K)	302
a, *b*, *c* (Å)	8.6902 (3), 16.6564 (5), 6.9636 (3)
β (°)	112.529 (5)
V (Å^3)	931.05 (7)
Z	4
Radiation type	Cu Kα
μ (mm^{-1})	1.11
Crystal size (mm)	0.10 × 0.04 × 0.02
T_{min}, T_{max}	0.152, 1.000
# measured, independent and observed [$I > 2\sigma(I)$] reflections	6025, 1826, 1406
R_{int}	0.025
$(\sin \theta/\lambda)_{max}$ (Å^{-1})	0.631
$R[F^2 > 2\sigma(F^2)]$, $wR(F^2)$, S	0.044, 0.144, 1.09
No. of reflections	1826
No. of parameters	179
$\Delta\rho_{max}$, $\Delta\rho_{min}$ (e Å^{-3})	0.19, −0.29
CCDC number	2370899

The single crystal data were collected at 302 K using a XtaLAB Synergy diffractometer, equipped with a HyPix detector. Unit cell refinement and data reduction were performed using CrysAlisPro 1.171.41.98a. Structures were solved by direct methods using SHELXT and refined by full-matrix least-squares on F^2 with anisotropic displacement parameters for the non-H atoms using ShelXL and Olex2. Absorption correction was performed

based on multi-scan procedure.[1–4] CCDC Deposition Number 2370899 contains the supplementary crystallographic data for this paper. These data are provided free of charge by the Cambridge Crystallographic Data Centre.

Textural and compositional data

***Table S2**. Composition and textural properties of the materials.*

Catalyst	C^a (wt.%)	N^a (wt.%)	H^a (wt.%)	C/N (-)	Ni^b (wt.%)	$S_{BET}{}^c$ ($m^2\ g^{-1}$)
CN_x	31.90	48.75	2.43	0.65	-	187
$Ni_1@CN_x$	32.59	52.62	2.32	0.62	0.48	166

[a]CHN combustion analysis. [b]ICP-OES. [c]BET method applied on the adsorption branch of the N_2 isotherm in the $0.05 < p/p_0 < 0.3$ range.

Quantitative data obtained from ICP-OES confirmed the successful introduction of Ni species within the porous CN_x frameworks, resulting in a metal loading of 0.48 wt.%. For both the Ni SAC and metal-free support, the nitrogen physisorption measurements revealed BET specific surface areas higher than those typically observed for bulk carbon nitride or exfoliated nanosheets.[5] The enhanced surface areas exhibited by the catalysts stemmed from the employment of a hard silica template in the synthesis process, subsequently removed through treatment with an etching agent, resulting in the production of mesopores in the three-dimensional framework of the CN_x support. Moreover, these quantitative data demonstrated that the incorporation of Ni species within the CN_x support did not disrupt the mesoporous nature of the prepared materials, consistently with data previously reported in the literature.[6,7]

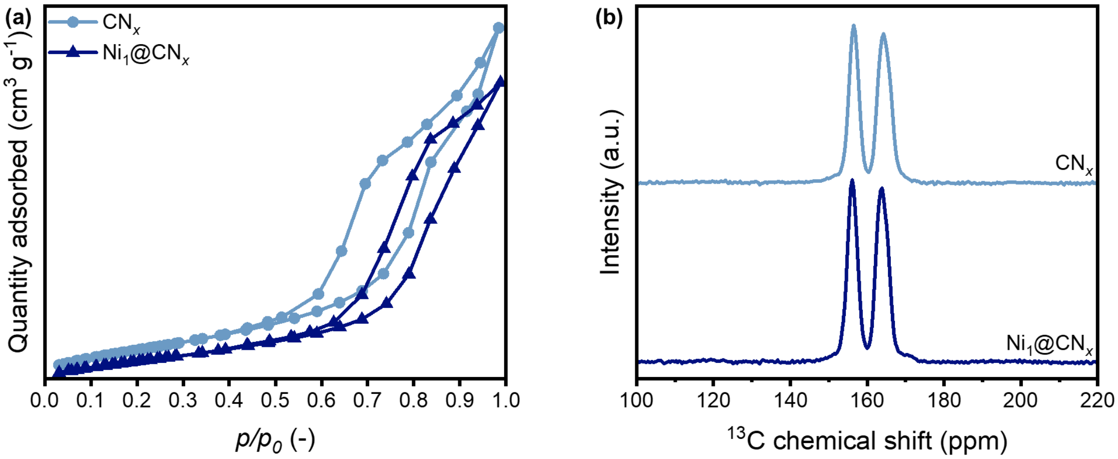

Figure S1. (a) *N_2 physisorption isotherms and* ***(b)*** *^{13}C solid-state NMR spectra of the metal-free CN_x support and $Ni_1@CN_x$ single-atom catalyst.*

FT-IR spectroscopy data

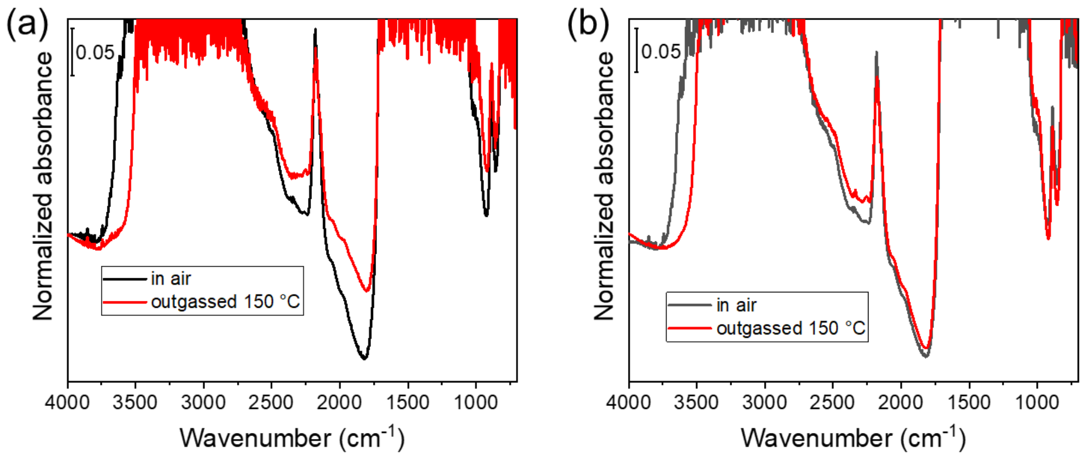

Figure S2. *FT-IR spectra of* ***(a)*** *the CN_x support and* ***(b)*** *the $Ni_1@CN_x$ catalyst, in air (black curves) and after outgassing at 150 °C for 1 h (red curves).*

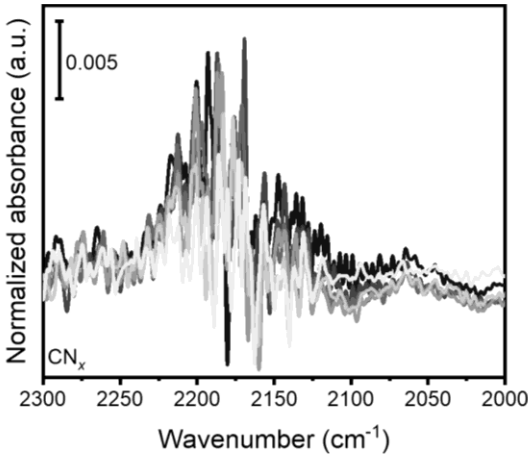

Figure S3*. FT-IR spectra of CO adsorption at 100 K at progressively decreasing partial pressures (from black to light grey) on the CN_x support outgassed at 150 °C for 1 h. The spectrum of the sample before CO adsorption was subtracted from all spectra.*

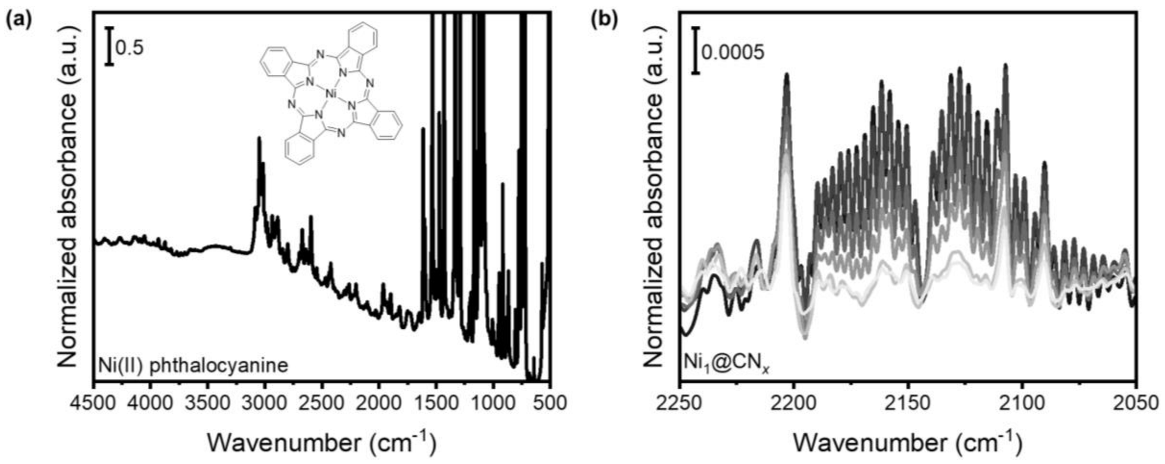

Figure S4. ***(a)*** *FT-IR spectrum of Ni(II) phthalocyanine in air.* ***(b)*** *FT-IR spectra of CO adsorption at 100 K on Ni(II) phthalocyanine at progressively decreasing partial pressures (from black to light grey). The signal related to CO adsorption on Ni is the sharp peak at 2203 cm^{-1}, while the other signals at lower wavenumbers are due to roto-vibrational transitions of CO in the gas phase. The spectrum of the sample before CO adsorption was subtracted from all spectra. The 2D structure of Ni(II) phthalocyanine is reported as an inset in* ***Figure S4a****.*

ToF-SIMS data

***Table S3**. Peak list of selected ions from the positive mass spectra of CN_x and $Ni_1@CN_x$.*

Average mass centre	Assignment	Normalized area in CN_x	Normalized area in $Ni_1@CN_x$
11.9985	C^+	0.11	0.06
14.0013	N^+	0.01	0.01
17.0257	NH_3^+	0.16	0.17
18.0342	NH_4^+	0.74	1.21
27.0087	CHN^+	0.15	0.14
27.0216	$C_2H_3^+$	0.22	0.32
28.0179	CH_2N^+	1.91	1.91
29.0378	$C_2H_5^+$	0.29	0.46
30.0331	CH_4N^+	0.12	0.23
30.9965	CF^+	0.3	0.04
31.0167	CH_3O^+	0.06	0.12
39.0198	$C_3H_3^+$	0.16	0.23
43.0292	$CH_3N_2^+$	9.88	9.02
53.0097	$C_2HN_2^+$	0.91	0.89
57.9327	$^{58}Ni^+$	-	0.21
59.9294	$^{60}Ni^+$	-	0.08
61.9291	$^{62}Ni^+$	-	0.01
68.0285	$C_4H_4O^+$	15.89	15.53
84.9412	$^{58}NiCNH^+$	-	0.11
86.9359	$^{60}NiCNH^+$	-	0.04
88.9339	$^{62}NiCNH^+$	-	0.01
93.0143	$SiC_5H_5^+$	2.92	2.94
110.0473	$C_3H_4N_5^+$	1.95	1.80
118.0095	$C_3H_4NO_4^+$	0.56	0.60
124.9428	$^{58}NiC_3N_2H_3^+$	-	0.183
126.9390	$^{60}NiC_3N_2H_3^+$	-	0.077

127.0638	$C_6H_9NO_2^+$	1.486	0.905
128.9367	$^{62}NiC_3N_2H_3^+$	-	0.013
130.9761	$C_4O_4F^+$	0.369	0.044
152.0602	$C_7H_8N_2O_2^+$	1.88	1.605
177.0479	$C_9H_7NO_3^+$	2.839	2.499

***Table S4**. Peak list of selected ions from the negative mass spectra of CN_x and Ni_1@CN_x.*

Average mass centre	Assignment	Normalized area in CN_x	Normalized area in Ni_1@CN_x
18.999	F^-	0.0027	0.0041
26.008	CN^-	0.1801	0.2114
40.013	CN_2^-	0.0181	0.0219
41.021	CHN_2^-	0.0180	0.0219
66.034	$C_3H_2N_2^-$	0.3418	0.3754
67.027	$C_2HN_3^-$	0.0127	0.0153
90.028	$C_2H_4NO_3^-$	0.0042	0.0070
92.031	$C_4H_3N_3^-$	0.0032	0.036
93.041	$C_4H_3N_3^-$	0.0057	0.0066

To investigate the coordinative environment of the Ni species within the CN_x pores, we conducted ToF-SIMS measurements. As observed in **Tables S3** and **S4**, in the ToF-SIMS spectra of both the pristine support and Ni_1@CN_x, we observed fragments expected from a CN_x structure, characterized by the general formula $C_wN_xH_y$ (with w = 0-9, x = 1-4, y = 0-7). Furthermore, we detected traces of impurities – including oxygen and fluorine – whose presence derives from SACs synthesis procedure and support/sample handling under atmospheric conditions. The ToF-SIMS spectra of metal-free CN_x and Ni SAC exhibited very similar fingerprint spectra, differing mainly in the regions displaying signals arising from the presence of metal ions in the catalyst fragments. Among the detected ions originating from the Ni SAC, the most frequently observed Ni-containing charged species included Ni^+, $NiCNH^+$, and $NiC_3N_2H_3^+$. ToF-SIMS results suggested as a preliminary inference that Ni active species may be predominantly coordinated to nitrogen atoms within the CN_x framework.

Supporting DFT calculations

CO on Ni@HP-CN$_x$ (periodic model, VASP calculations, PBE+U+D3 functional)

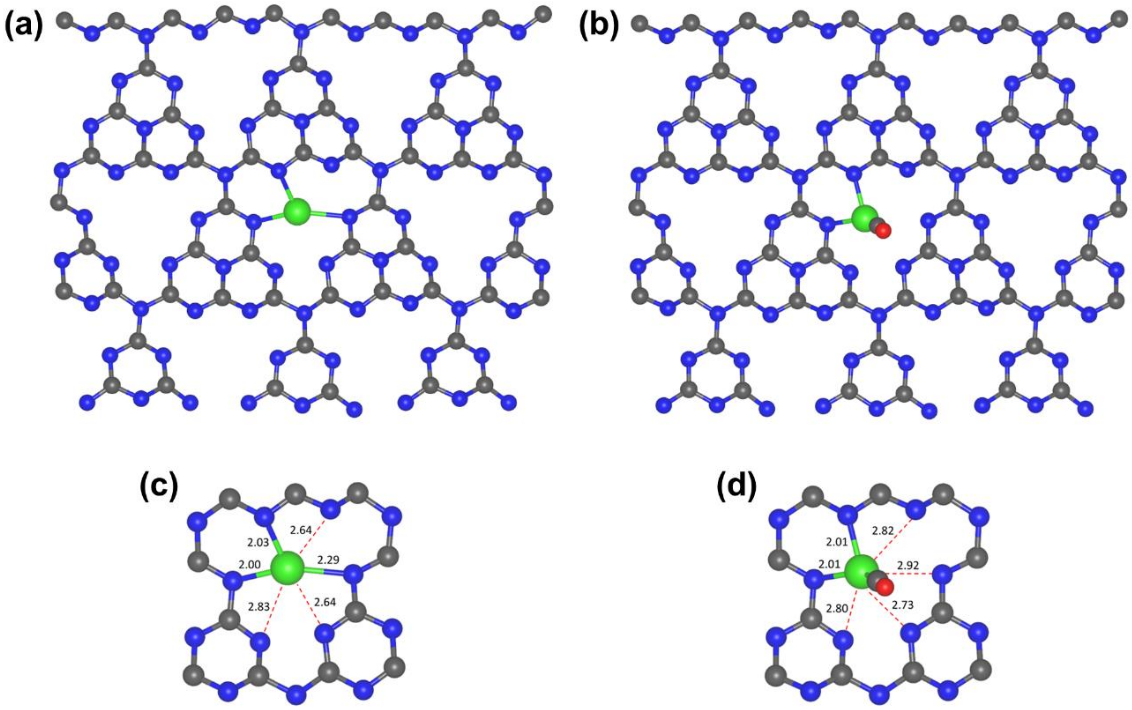

Figure S5. ***(a)*** *Top view of the DFT-optimized Ni@HP-CN$_x$ periodic structure.* ***(b)*** *Top view of the DFT-optimized Ni@HP-CN$_x$ periodic structure with a CO molecule adsorbed on the Ni atom. Zoom of the heptazine pore with the distance between the metal and the N atoms in the absence* ***(c)*** *and presence* ***(d)*** *of CO. VASP calculations, PBE+U+D3 functional. Colour code: dark grey = carbon, blue = nitrogen, green = Ni, red = oxygen.*

Table S5. *Properties of the DFT-optimized Ni@HP-CN$_x$ structure and its complex with CO^a (VASP calculations, PBE+U+D3 functional).*

	***r*(Ni-N) (Å)**	**Δ*E*(CO) (eV)**	***r*(Ni-C) (Å)**	***r*(C-O) (Å)**	**Δ*ω*(CO) (cm^{-1})**
Ni@HP-CN$_x$	2.00, 2.03, 2.29, 2.64	-	-	-	-
CO-Ni@HP-CN$_x$	2.01, 2.01	–1.06	1.79	1.16	–75

^{a}The CO frequency in gas phase with the PBE+D3 functional is 2125.5 cm^{-1}.

Test calculations of the accuracy of CO vibrational frequencies (Molecular models, Gaussian09 calculations, PBE0+D3 functional)

The computational approach adopted here (Gaussian09 calculations, PBE0+D3 functional, 6-31++G(d, p) basis set) was benchmarked by computing some Ni(CO) complexes with experimental data available in the literature: Ni(CO), $[Ni(CO)]^{1+}$, Ni(CO)Cl, $Ni(CO)Cl_2$. The results (**Table S6**) showed that this approach is able to describe the vibrational properties of the Ni(CO) complexes (no scaling of the computed frequencies is applied). The measured positive CO vibrational shift associated with $[Ni^{+I}(CO)]^{1+}$ and $Ni^{+II}(CO)Cl_2$ species was properly reproduced.

Table S6. *Comparison of computed and experimental properties of representative Ni-CO complexes (Gaussian09 calculations, PBE0+D3 functional, 6-31++G(d, p) basis set).*

	**Δ*E*(CO) (eV)**	***r*(Ni-C) (Å)**	***r*(C-O) (Å)**	**Δ*ω*(CO)[a] (cm^{-1})**	**Δ*ω*(CO)[b] (cm^{-1})**
Ni(CO)	–1.49	1.67	1.16	–119	–149[22]
$[Ni^{+I}(CO)]^{1+}$	–1.79	1.87	1.13	+86	+63[22]
$[Ni^{+II}(CO)]^{2+}$	–1.43	1.89	1.11	+178	-
$Ni^{+I}(CO)Cl$	–1.93	1.80	1.14	–15	–24[23]
$Ni^{+II}(CO)Cl_2$	–0.74	1.98	1.13	+50	+47[23]

[a]DFT-predicted. [b]Experimental values from the literature.

CO adsorption on Ni phthalocyanine, Ni@4N-Pc (molecular model, Gaussian09 calculations, PBE0+D3 functional)

For the Ni phthalocyanine complex (Ni@4N-Pc), we performed high-quality DFT calculations using a hybrid functional, including dispersion contributions, and Gaussian-type orbitals basis sets (Gaussian09 calculations, PBE0+D3 hybrid exchange-correlation functional, 6-31++G(d, p) basis set), as shown in **Figure S6** and **Table S7**.

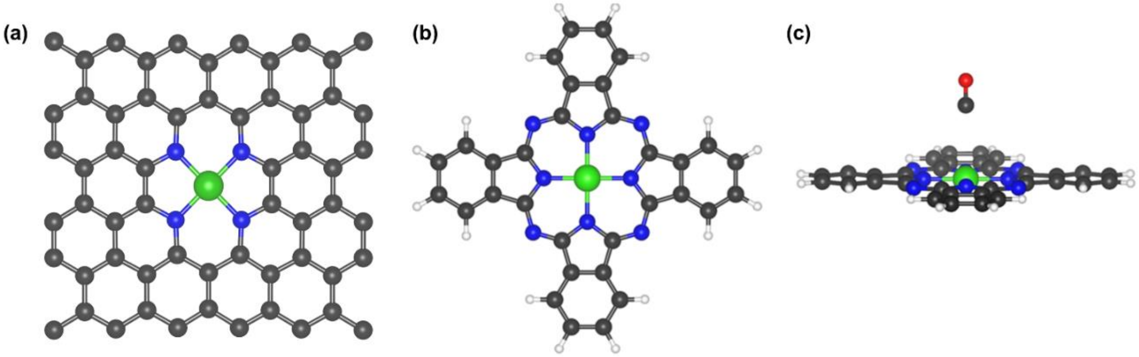

Figure S6. ***(a)*** *Top view of the Ni@4N-Gr extended periodic structure (VASP calculations).* ***(b)*** *Top view of the Ni phthalocyanine [Ni@4N-Pc] complex.* ***(c)*** *Side view of the Ni phthalocyanine [Ni@4N-Pc] complex with a CO molecule adsorbing on the Ni atom. Gaussian09 calculations, PBE0+D3 functional, 6-31++G(d, p) basis set. Colour code: dark grey = carbon, blue = nitrogen, green = nickel, red = oxygen, white = hydrogen.*

Table S7. *Properties of [Ni@4N-Pc]$^{n+}$ complex interacting with CO with n = 0, 1, 2 (Gaussian09 calculations, PBE0+D3 functional, 6-31++G(d, p) basis set).*[a]

	***r*(Ni-N) (Å)**	**Δ*E*(CO) (eV)**	***r*(Ni-C) (Å)**	***r*(C-O) (Å)**	**Δ*ω*(CO) (cm^{-1})**
[Ni@4N-Pc]^{0}(CO)	1.91	–0.12	3.38	1.13	+1
[Ni@4N-Pc]$^{1+}$(CO)	1.90	–0.15	3.36	1.13	+15
[Ni@4N-Pc]$^{2+}$(CO)	1.89	–0.17	3.34	1.13	+27

[a]The results reported here are also consistent with a recently reported DFT study carried out on the same Ni-based phthalocyanine system.[8]

CO adsorption on Ni-heptazine ligand complexes, Ni@HL-CN$_x$ (molecular models, Gaussian09 calculations, PBE0+D3 functional)

The nature of the Ni-heptazine ligand complexes (Ni@HL-CN$_x$) was studied with the Gaussian09 code. In the [Ni@HL-CN$_x$]^0 neutral complex, Ni is coordinated to two nitrogen atoms, **Figures S7a** and **S7d**, and CO strongly binds to the Ni species resulting in a red-shifted frequency of –215 cm^{-1} (**Table S8**). When the complex carries a positive charge, [Ni@4N-heptazine]$^{1+}$, Ni interacts with three nitrogen atoms through shorter bonds and one nitrogen atom with a longer bond (**Figures S7b** and **S7e** and **Table S8)**; in this case, CO remains strongly bound to the Ni site, but the corresponding frequency exhibits a negative shift of –55 cm^{-1}. These results deviated from the experimental data both in terms of the coordination environment of the Ni atom (2 or 3 neighbour instead of 4) and of CO vibrational shift, which shows an opposite sign compared to the one observed experimentally in FT-IR measurements.

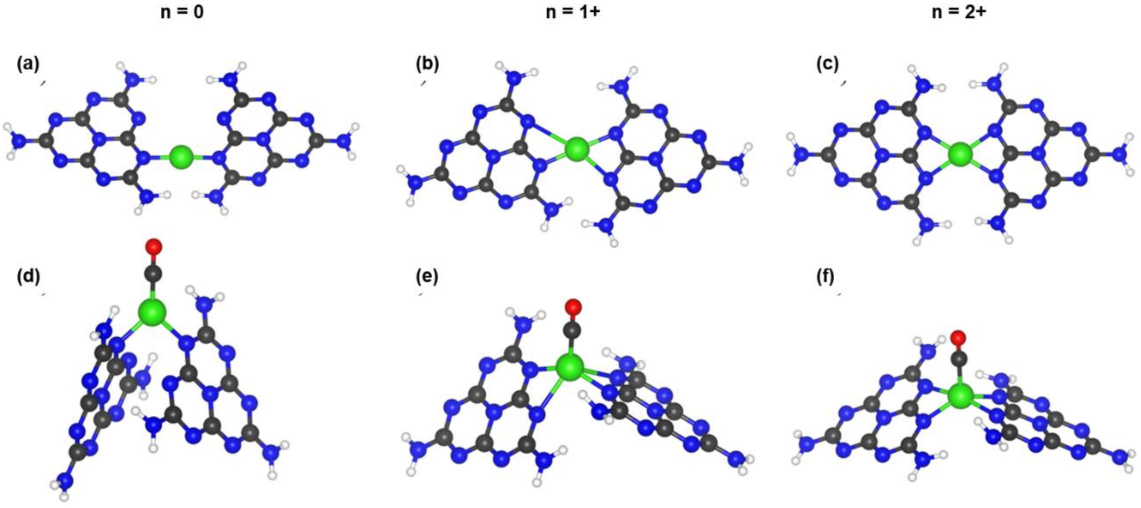

Figure S7*. Top view of the [Ni@HL-CN$_x$]$^{n+}$ complexes with **(a)** n = 0, **(b)** n = 1, and **(c)** n = 2. Spin population (SP) and Mulliken charge (q) of the three complexes: (a) [Ni@HL-CN$_x$]^0 SP = 0.95, q = +0.54 |e|; (b) [Ni@HL-CN$_x$1^{1+} SP = 0.99, q = +0.55 |e|; (c) [Ni@HL-CN$_x$]$^{2+}$ SP = 1.75, q = +1.05 |e|.*

*Side view of the [Ni@HL-CN$_x$]$^{n+}$ complexes with **(d)** n = 0, **(e)** n = 1, and **(f)** n = 2, with a CO molecule adsorbed on the Ni atom. Gaussian09 calculations, PBE0+D3 functional, 6-31++G(d, p) basis set. Colour code: dark grey = carbon, blue = nitrogen, green = nickel, red = oxygen, white = hydrogen.*

Table S8. *Properties of [Ni@*HL-CN$_x$*]$^{n+}$ complexes (n = 0, 1, 2) interacting with CO where all the peripheral atoms of the heptazine molecule being N atoms (Gaussian09 calculations, PBE0+D3 functional, 6-31++G(d, p) basis set).*

	***r*(Ni-N) (Å)**	**Δ*E*(CO) (eV)**	***r*(Ni-C) (Å)**	***r*(C-O) (Å)**	**Δ*ω*(CO) (cm^{-1}) DFT(PBE0+D3)**
Small model					
[Ni@HL-CN$_x$]^0	1.86, 2.99	–2.15	1.69	1.17	–215
[Ni@HL-CN$_x$]$^{1+}$	1.93, 1.99, 2.20, 2.79	–1.04	1.81	1.14	-55
[Ni@HL-CN$_x$]$^{2+}$ (S)[a]	1.92, 1.95	–0.84	1.83	1.13	+42
[Ni@HL-CN$_x$]$^{2+}$ (T)[a]	2.00, 2.04	–1.03	2.00	1.13	+77
Large model					
[Ni@(HL-CN$_x$)$_3$]$^{2+}$ (T)[a,b]	1.94, 2.09	–1.06	1.99	1.13	+79

[a]S = singlet, T = triple. [b]A 6-31G(d, p) basis set has been used, instead of the more extended 6-31++G(d, p) basis set adopted for the other calculations. We made sure that this has no major effects on the results by performing test calculations with the smaller basis set.

The model [Ni@HL-CN$_x$]$^{2+}$ complex depicted in **Figures S7c** and **S7f** is relatively small and may not fully capture the complexity of the heptazine ligands. Therefore, we explored a new structure where three heptazine units are linked together to form a sort of trimeric structure, [Ni@(HL-CN$_x$)$_3$]$^{2+}$, creating a more rigid framework that could affect the local structure of the Ni centres (**Figure S8** and **Table S8**). Only the 2+ charged state and the triplet configuration of the [Ni@(HL-CN$_x$)$_3$] complex were considered. The key finding was that even with the "trimeric" heptazine ligands, the structural model remained similar to that described above for the smaller [Ni@HL-CN$_x$] complex. Two Ni-N bond distances measure 1.94 Å, and two *ca.* 0.15 Å longer at 2.09 Å (2.02 Å in average, similar to the smaller model). CO binds to the Ni site by an adsorption energy of –1.06 eV, and the CO frequency shift in the FT-IR spectra is +79 cm^{-1} (compared to +77 cm^{-1} in the smaller [Ni@(HL-CN$_x$] model). Additionally, the Ni-CO distance of 1.99 Å is nearly identical to that in the smaller complex (**Table S8**).

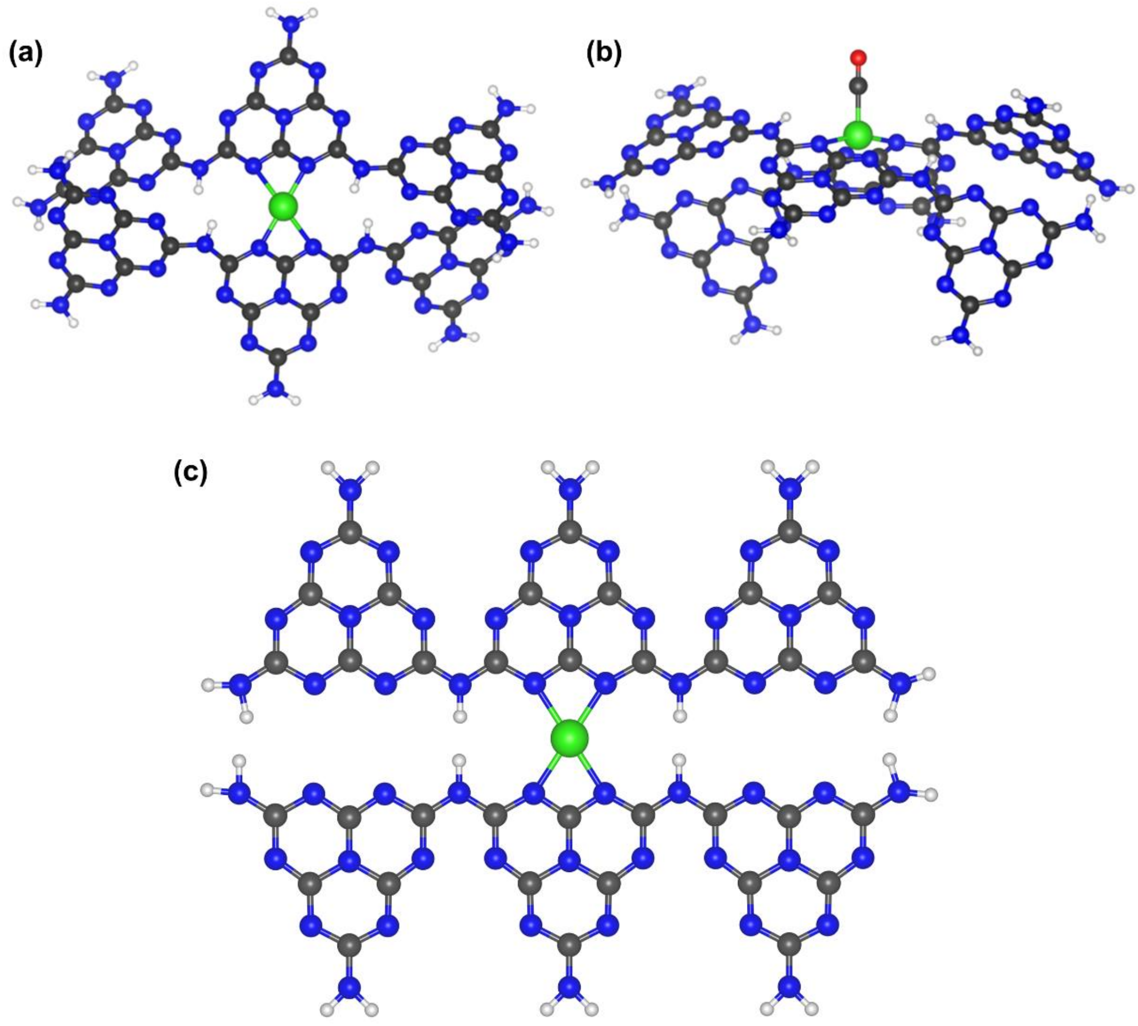

Figure S8. ***(a)*** *Top view of the large model of the* $[Ni@(HL\text{-}CN_x)_3]^{2+}$ *complex. Spin population (SP) and Mulliken charge (q) of* $[Ni@(HL\text{-}CN_x)_3]^{2+}$*: SP = 1.74, q = +1.02 |e|.* ***(b)*** *Side view of the* $[Ni@(HL\text{-}CN_x)_3]^{2+}$ *complex with a CO molecule adsorbed on the Ni atom. (c) Top view of the extended Ni@HL-CN*$_x$ *surface. Gaussian09 calculations, PBE0+D3 functional, 6-31G(d, p) basis set. Colour code: dark grey = carbon, blue = nitrogen, green = nickel red = oxygen, white = hydrogen.*

Ni-heptazine ligand complexes, Ni@HL-CN (periodic models, VASP calculations, PBE+U+D3 functional)

The results obtained with molecular models (using Gaussian09 code) were validated against periodic models (using VASP code), with particular focus on the structural aspects and the coordination environment of the Ni ion. The quality of the periodic results differs from the molecular calculations for two main reasons. First, periodic

calculations, involving charged supercells require a neutralizing background charge. While this works well for bulk systems, it is more complex when adsorption phenomena are involved due to the need for a vacuum layer along the non-periodic direction. We also considered neutral supercells with a counterion to compensate for the Ni^{2+} ion's charge. Second, to mitigate the high computational cost of hybrid functionals when plane wave basis sets are adopted, we used the self-interaction corrected PBE+U+D3 approach, which strikes d a good balance between computational cost and accuracy. Constructing periodic models of triazine ligands allowed us to model a solid system where spatial constraints might result in modified local bonding modes.

We first examined the same $[Ni@HL\text{-}CN_x]^{2+}$ molecular complex previously described (**Table S8**). The resulting structure (**Figure S9a**) resembles that obtained with the Gaussian09 code (**Figures S7c** and **S7f**), with the Ni ion coordinated to four nitrogen atoms in a distorted square-planar coordination. The main difference is that the ground state is singlet and that the Ni-N distances are slightly longer (*ca.* 1.97 Å) than in the molecular calculations (1.92–1.95 Å, see **Table S8)**. This may be due to the use of a different functional (PBE+U+D3 versus PBE0+D3) or the charged supercell. In a different model, the charge of the Ni^{2+} ion is compensated by a carbonate ion, $(CO_3)^{2-}$, directly bound to the Ni site (**Figure S9c**). We observed an additional elongation of the Ni-N distances, probably because the net charge on Ni is lower than in the previous example: more specifically, two Ni-N distances increase to 2.00 Å and two to 2.14 Å, averaging 2.07 Å.

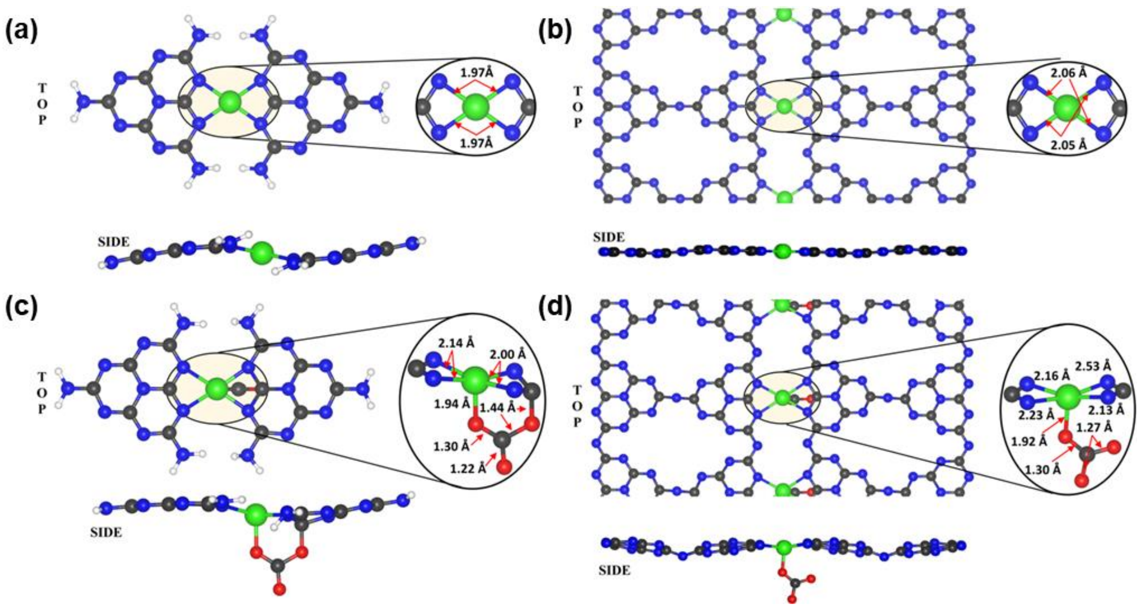

Figure S9. *Properties of [Ni@4N-Triazine]$^{2+}$ complexes from periodic calculations.* ***(a)*** *Structure of [Ni@HL-CN$_x$]$^{2+}$ as a molecular complex.* ***(b)*** *Structure of [Ni@HL-CN$_x$]$^{2+}$ as a periodic complex.* ***(c)*** *Structure of [Ni@HL-CN$_x$]$^{2+}$[CO$_3$]$^{2-}$ as a molecular complex.* ***(d)*** *Structure of [Ni@HL-CN$_x$]$^{2+}$[CO$_3$]$^{2-}$ as a periodic complex. VASP calculations, PBE+U+D3 functional, plane waves basis set. Colour code: dark grey = carbon, blue = nitrogen, green = nickel, red = oxygen, white = hydrogen.*

In the next step, a fully periodic model was generated and computed with either charged supercells (**Figure S9b**) or a compensating carbonate ion (**Figure S9d**). These new models were fully optimized. In the charged complex, the Ni atom remains tetracoordinated in a nearly square-planar geometry, and the structure of the support forms an almost flat two-dimensional layer (**Figure S9b**). The Ni-N distances are *ca.* 0.08 Å longer than those in the molecular model computed at the same level (**Figure S9a**), likely due to some tensile strain introduced by the boundary conditions of the supercell. When the neutral complex is considered, the presence of the carbonate ion interacting with the Ni centre induces a substantial distortion (**Figures S9c-d**). Ni is bound to three nitrogen atoms with elongated distances (2.13–2.23 Å), while one nitrogen atom is significantly far (with a Ni-N distance of 2.53 Å). These results led us to conclude that in the periodic models, despite the spatial constraints imposed on the triazine ligands by the formation of a solid system, the local coordination environment of the Ni^{2+} ion remains largely consistent with that observed in the molecular models with a slight elongation of the Ni-Ni distances, which

may have a structural origin or be due to the approach used). The inclusion of a carbonate ion bound to the Ni centre to compensate for the charge results in local distortions, suggesting that the counter-ions are probably localized at different sites in the support materials rather than directly interacting with the Ni sites.

X-ray absorption spectroscopy data

Table S9. *Convolution parameters for FDMNES simulations with DFT-based structures (Ni@HP-CN$_x$, Ni@4N-Pc, Ni@HL-CN$_x$ in the triplet and single states).*

**Structure**	**Gamma hole (eV)**	**Gamma max (eV)**	E_{cent} **(eV)**	E_{large} **(eV)**	E_{cut} **(eV)**	E_{start} **(eV)**
Ni@HP-CN$_x$	1.44	15	30.0	30.0	–1.561	–20
Ni@4N-Pc	1.44	15	30.0	30.0	–1.561	–20
Ni@HL-CN$_x$ (T)	1.44	15	30.0	30.0	–1.561	–20
Ni@HL-CN$_x$ (S)	1.44	15	30.0	30.0	–1.561	–20

Table S10. *First-shell EXAFS fitting parameters for Ni(II) phthalocyanine model compound used to fit experimental EXAFS spectra. R_0 values indicate the starting Ni-N distances in DFT models.*

**Structure**	S_0^2	E_0 **(eV)**	R_0 **(Å)**	ΔR_N **(Å)**	σ^2_N **(Å^2)**	***R* factor**
**Ni phthalocyanine**	0.77 ± 0.39	-7 ± 6	1.95 [N$_N$=4]	-0.08 ± 0.03	0.001 ± 0.005	0.084

Table S11. *First-shell EXAFS fitting parameters for four DFT-based structures used to fit experimental EXAFS spectra. Underlined values were fixed in the fit. R_0 values indicate the starting Ni-N distances in DFT models and N_N the number of N atoms for each distance.*

**Structure**	S_0^2	E_0 **(eV)**	R_0 **(Å)**	ΔR_N **(Å)**	σ^2_N **(Å^2)**	***R* factor**
Ni@HP-CN$_x$	0.8	2 ± 10	2.00 [N$_N$=1]<br>2.04 [N$_N$=1]<br>2.27 [N$_N$=1]	-0.12 ± 0.06	-0.0001 ± 0.005	0.308
Ni@4N-Pc	0.8	-6 ± 4	1.89 [N$_N$=4]	-0.02 ± 0.04	0.006 ± 0.003	0.088
Ni@HL-CN$_x$ (T)	0.8	-4 ± 5	2.01 [N$_N$=2]<br>2.04 [N$_N$=2]	-0.16 ± 0.04	0.006 ± 0.002	0.095
Ni@HL-CN$_x$ (S)*	0.8	-4 ± 5	1.93 [N$_N$=4]	-0.07 ± 0.03	0.008 ± 0.002	0.065

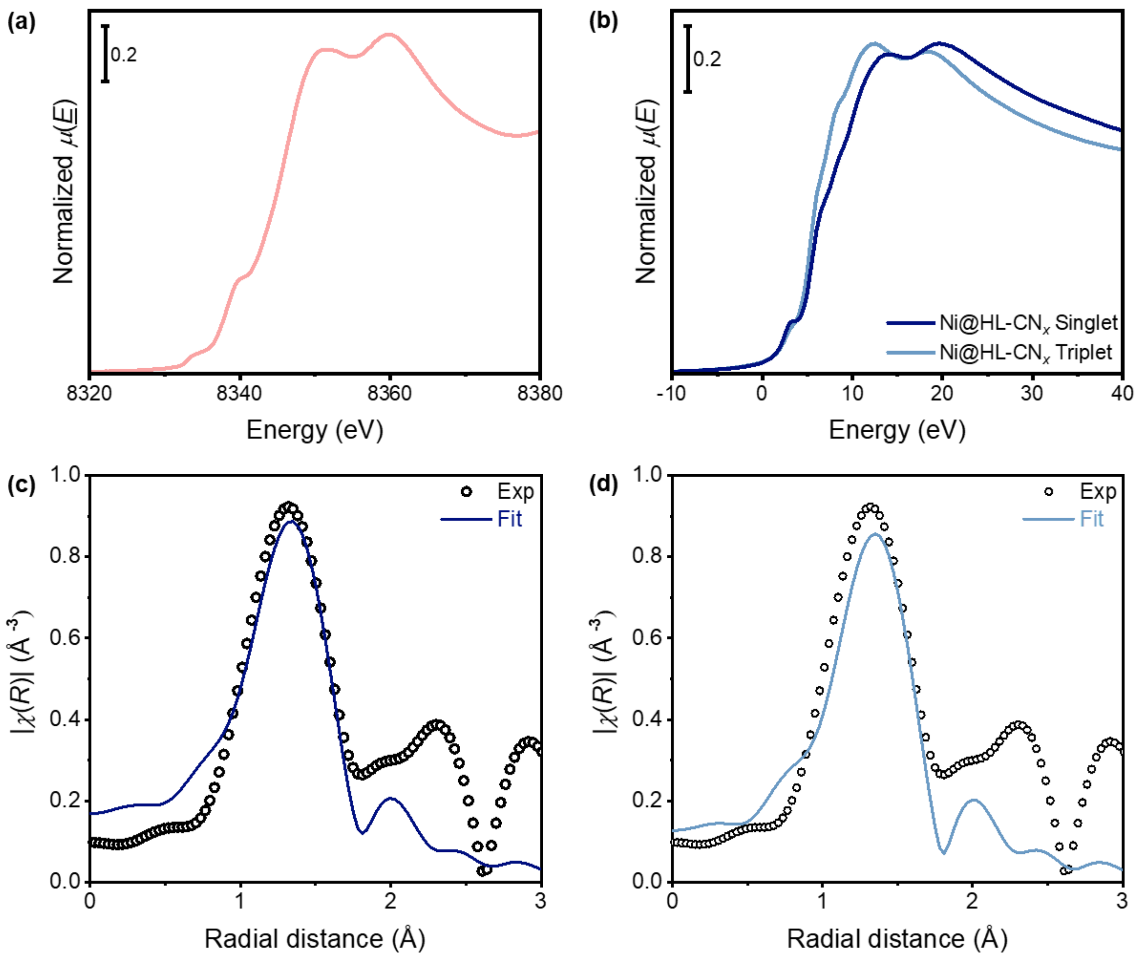

Figure S10. (a) *Experimental Ni K edge XANES spectrum of* Ni_1*@*CN_x*, and* ***(b)*** *FDMNES simulations with two Ni@HL-*CN_x *(singlet and triplet) DFT structures of Ni catalyst at R = 6 Å. Bottom panels present the phase-uncorrected,* k^2*-weighted Fourier-transformed EXAFS data plotted as a function of radial distance R, showing the first-shell EXAFS fitting performed with the corresponding DFT structures:* ***(c)*** *HL-*CN_x *singlet and* ***(d)*** *HL-*CN_x *triplet.*

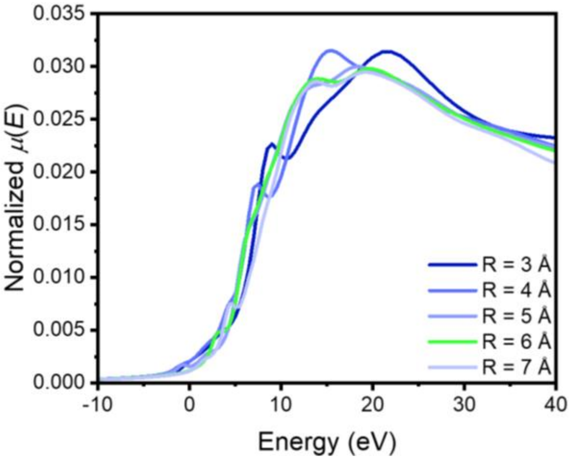

Figure S11. *FDMNES simulations with Ni@HL-CN_x singlet DFT structure of Ni SAC conducted considering radii centred on the Ni atom with diameter ranging 3-7 Å to assess convergence (reached at 6 Å, green plot).*

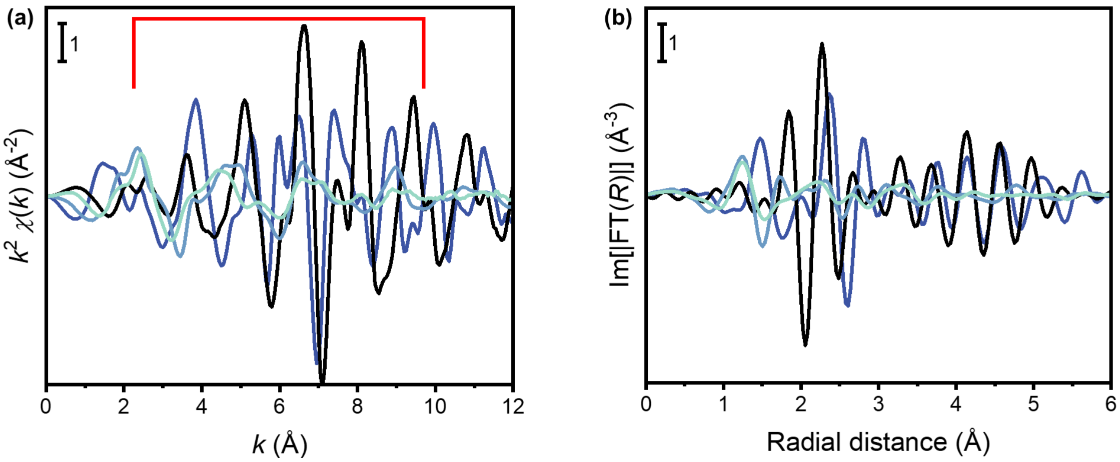

Figure S12. (a) *k^2-weighted $\chi(k)$ spectra and* ***(b)*** *imaginary part of phase-uncorrected FT-EXAFS spectra of Ni_1@CN_x, Ni(II) phthalocyanine, and nickel-containing reference materials. Colour code: black = Ni foil, dark blue = NiO, blue = Ni(II) phthalocyanine, and light blue = Ni_1@CN_x. The window represents the k range (2.5-10 $Å^{-1}$) in which the FT-EXAFS spectra are extracted.*

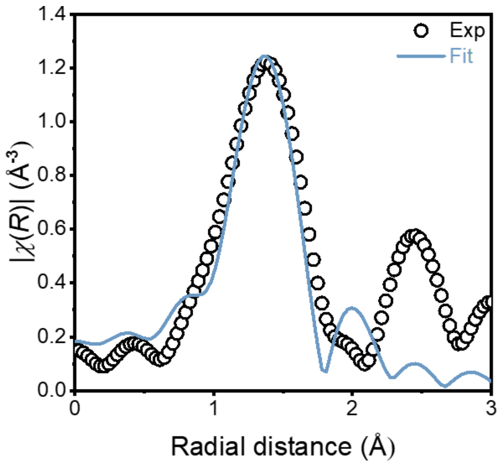

Figure S13. *First-shell EXAFS fitting performed for phase-uncorrected FT-EXAFS spectra of Ni(II) phthalocyanine model compound. The results of the fit are summarized in Table S10.*

CIF file for $[Ni@HL\text{-}CN_x]^{2+}$ (T) as a molecular complex with Gaussian

```
data_image0
_chemical_formula_structural       C12N18H12NiCHCH
_chemical_formula_sum              "C14 N18 H14 Ni1"
loop_
 _atom_site_type_symbol
 _atom_site_label
 _atom_site_symmetry_multiplicity
 _atom_site_Cartn_x
 _atom_site_Cartn_y
 _atom_site_Cartn_z
 _atom_site_occupancy
 C   C1     1.0  -4.30050  -1.24310  -0.46660  1.0000
 C   C2     1.0  -2.38110  -0.04880   0.28890  1.0000
 C   C3     1.0  -2.26680  -2.28630  -0.22550  1.0000
 C   C4     1.0  -2.36240   2.24070   0.69620  1.0000
 C   C5     1.0  -4.41020   1.17410  -0.04670  1.0000
 C   C6     1.0  -6.21600  -0.03870  -0.69810  1.0000
 C   C7     1.0   4.30050   1.24310  -0.46640  1.0000
 C   C8     1.0   2.38110   0.04880   0.28900  1.0000
 C   C9     1.0   2.26680   2.28630  -0.22530  1.0000
 C   C10    1.0   2.36230  -2.24070   0.69620  1.0000
 C   C11    1.0   4.41020  -1.17410  -0.04660  1.0000
 C   C12    1.0   6.21600   0.03870  -0.69790  1.0000
 N   N1     1.0  -3.68400  -0.03010  -0.05690  1.0000
 N   N2     1.0  -3.55330  -2.34320  -0.53610  1.0000
 N   N3     1.0  -1.63820  -1.15550   0.24090  1.0000
 N   N4     1.0  -1.70460   1.03650   0.65550  1.0000
 N   N5     1.0  -5.56800  -1.22600  -0.77100  1.0000
 N   N6     1.0  -5.67600   1.16330  -0.36750  1.0000
 N   N7     1.0  -7.50510  -0.04970  -0.98840  1.0000
 N   N8     1.0  -1.58920   3.30320   1.06270  1.0000
 N   N9     1.0  -1.52640  -3.38170  -0.36030  1.0000
 N   N10    1.0   3.68400   0.03010  -0.05670  1.0000
 N   N11    1.0   3.55340   2.34320  -0.53590  1.0000
 N   N12    1.0   1.63820   1.15550   0.24100  1.0000
 N   N13    1.0   1.70460  -1.03650   0.65550  1.0000
 N   N14    1.0   5.56800   1.22610  -0.77080  1.0000
 N   N15    1.0   5.67600  -1.16330  -0.36730  1.0000
 N   N16    1.0   7.50510   0.04970  -0.98810  1.0000
 N   N17    1.0   1.58920  -3.30330   1.06270  1.0000
 N   N18    1.0   1.52640   3.38170  -0.36010  1.0000
 H   H1     1.0  -8.03780   0.80730  -0.95610  1.0000
 H   H2     1.0  -7.95280  -0.91760  -1.24610  1.0000
 H   H3     1.0  -0.89190   3.11400   1.77210  1.0000
 H   H4     1.0  -2.07420   4.17890   1.21030  1.0000
 H   H5     1.0  -0.53140  -3.38180  -0.18320  1.0000
 H   H6     1.0  -1.97100  -4.21500  -0.71880  1.0000
 H   H7     1.0   8.03790  -0.80720  -0.95580  1.0000
 H   H8     1.0   7.95280   0.91760  -1.24580  1.0000
 H   H9     1.0   0.89180  -3.11400   1.77200  1.0000
 H   H10    1.0   2.07420  -4.17890   1.21030  1.0000
 H   H11    1.0   0.53140   3.38180  -0.18300  1.0000
 H   H12    1.0   1.97100   4.21510  -0.71850  1.0000
 Ni  Ni1    1.0   0.00000   0.00000   0.58790  1.0000
 C   C13    1.0  -3.69380   2.32940   0.34250  1.0000
 H   H13    1.0  -4.23780   3.26460   0.38610  1.0000
 C   C14    1.0   3.69380  -2.32940   0.34260  1.0000
 H   H14    1.0   4.23780  -3.26460   0.38610  1.0000
```

CIF file for $[Ni@HL\text{-}CN_x]^{2+}$ as a molecular complex with VASP

```
data_image0
_chemical_formula_structural       C12N20H12Ni
_chemical_formula_sum              "C12 N20 H12 Ni1"
_cell_length_a       20
_cell_length_b       15
_cell_length_c       15
_cell_angle_alpha    90
_cell_angle_beta     90
_cell_angle_gamma    90

_space_group_name_H-M_alt    "P 1"
_space_group_IT_number       1

loop_
  _space_group_symop_operation_xyz
  'x, y, z'

loop_
  _atom_site_type_symbol
  _atom_site_label
  _atom_site_symmetry_multiplicity
  _atom_site_fract_x
  _atom_site_fract_y
  _atom_site_fract_z
  _atom_site_occupancy
  C   C1        1.0  0.28574  0.42047  0.21139  1.0000
  C   C2        1.0  0.38721  0.50294  0.22471  1.0000
  C   C3        1.0  0.38796  0.34966  0.22747  1.0000
  C   C4        1.0  0.38847  0.65634  0.22772  1.0000
  C   C5        1.0  0.28609  0.58605  0.21161  1.0000
  C   C6        1.0  0.19176  0.50364  0.18978  1.0000
  C   C7        1.0  0.72090  0.58433  0.19549  1.0000
  C   C8        1.0  0.61936  0.50206  0.18125  1.0000
  C   C9        1.0  0.61874  0.65540  0.17883  1.0000
  C   C10       1.0  0.61809  0.34867  0.17889  1.0000
  C   C11       1.0  0.72045  0.41886  0.19545  1.0000
  C   C12       1.0  0.81471  0.50116  0.21746  1.0000
  N   N1        1.0  0.31993  0.50315  0.21888  1.0000
  N   N2        1.0  0.32182  0.34539  0.21937  1.0000
  N   N3        1.0  0.42372  0.42851  0.22595  1.0000
  N   N4        1.0  0.42391  0.57730  0.22609  1.0000
  N   N5        1.0  0.32233  0.66089  0.22012  1.0000
  N   N6        1.0  0.22107  0.42181  0.19758  1.0000
  N   N7        1.0  0.22136  0.58524  0.19787  1.0000
  N   N8        1.0  0.12661  0.50391  0.17195  1.0000
  N   N9        1.0  0.42275  0.27353  0.23715  1.0000
  N   N10       1.0  0.68659  0.50174  0.18776  1.0000
  N   N11       1.0  0.68488  0.65954  0.18728  1.0000
  N   N12       1.0  0.58297  0.57667  0.17956  1.0000
  N   N13       1.0  0.58256  0.42777  0.17975  1.0000
  N   N14       1.0  0.68413  0.34394  0.18706  1.0000
  N   N15       1.0  0.78551  0.58302  0.20959  1.0000
  N   N16       1.0  0.78508  0.41956  0.20955  1.0000
  N   N17       1.0  0.58299  0.27279  0.16929  1.0000
  N   N18       1.0  0.42345  0.73219  0.23792  1.0000
  N   N19       1.0  0.58418  0.73167  0.16934  1.0000
  N   N20       1.0  0.87984  0.50076  0.23518  1.0000
  H   H1        1.0  0.10291  0.56298  0.16237  1.0000
  H   H2        1.0  0.10279  0.44501  0.16205  1.0000
  H   H3        1.0  0.90387  0.55962  0.24473  1.0000
  H   H4        1.0  0.90341  0.44155  0.24466  1.0000
  H   H5        1.0  0.46997  0.72963  0.26498  1.0000
  H   H6        1.0  0.39633  0.78898  0.24635  1.0000
  H   H7        1.0  0.61183  0.78818  0.16167  1.0000
  H   H8        1.0  0.53770  0.73007  0.14203  1.0000
  H   H9        1.0  0.46926  0.27579  0.26438  1.0000
  H   H10       1.0  0.39539  0.21689  0.24540  1.0000
  H   H11       1.0  0.61012  0.21584  0.16171  1.0000
  H   H12       1.0  0.53655  0.27513  0.14172  1.0000
  Ni  Ni1       1.0  0.50334  0.50260  0.20273  1.0000
```

CIF file for $[Ni@HL\text{-}CN_x]^{2+}$ as a periodic complex with VASP

```
data_image0
_chemical_formula_structural       C12N17Ni
_chemical_formula_sum              "C12 N17 Ni1"
_cell_length_a       15.5927
_cell_length_b       6.8992
_cell_length_c       19.7814
_cell_angle_alpha    89.9612
_cell_angle_beta     90.1939
_cell_angle_gamma    90.2346

_space_group_name_H-M_alt    "P 1"
_space_group_IT_number       1

loop_
  _space_group_symop_operation_xyz
  'x, y, z'

loop_
  _atom_site_type_symbol
  _atom_site_label
  _atom_site_symmetry_multiplicity
  _atom_site_fract_x
  _atom_site_fract_y
  _atom_site_fract_z
  _atom_site_occupancy
  C   C1        1.0  0.25191  0.38019  0.09405  1.0000
  C   C2        1.0  0.38556  0.56060  0.09110  1.0000
  C   C3        1.0  0.38962  0.22436  0.09158  1.0000
  C   C4        1.0  0.38995  0.89668  0.09178  1.0000
  C   C5        1.0  0.25220  0.73889  0.09449  1.0000
  C   C6        1.0  0.12827  0.55900  0.09467  1.0000
  C   C7        1.0  0.83514  0.73837  0.08394  1.0000
  C   C8        1.0  0.70155  0.55796  0.08753  1.0000
  C   C9        1.0  0.69744  0.89422  0.08689  1.0000
  C   C10       1.0  0.69716  0.22186  0.08710  1.0000
  C   C11       1.0  0.83493  0.37957  0.08429  1.0000
  C   C12       1.0  0.95879  0.55959  0.08322  1.0000
  N   N1        1.0  0.29760  0.55985  0.09229  1.0000
  N   N2        1.0  0.30255  0.22477  0.09321  1.0000
  N   N3        1.0  0.43272  0.39947  0.09065  1.0000
  N   N4        1.0  0.43297  0.72217  0.09077  1.0000
  N   N5        1.0  0.30292  0.89492  0.09373  1.0000
  N   N6        1.0  0.16773  0.38440  0.09693  1.0000
  N   N7        1.0  0.16807  0.73378  0.09774  1.0000
  N   N8        1.0  0.04364  0.55993  0.08830  1.0000
  N   N9        1.0  0.43491  0.06114  0.09071  1.0000
  N   N10       1.0  0.78950  0.55866  0.08611  1.0000
  N   N11       1.0  0.78450  0.89383  0.08478  1.0000
  N   N12       1.0  0.65439  0.71904  0.08799  1.0000
  N   N13       1.0  0.65415  0.39636  0.08811  1.0000
  N   N14       1.0  0.78420  0.22362  0.08530  1.0000
  N   N15       1.0  0.91927  0.73409  0.08075  1.0000
  N   N16       1.0  0.91907  0.38477  0.08116  1.0000
  N   N17       1.0  0.65216  0.05743  0.08803  1.0000
  Ni  Ni1       1.0  0.54358  0.55926  0.08942  1.0000
```

References of the Supporting Information

(1) Sheldrick, G. M. *SHELXT* – Integrated Space-Group and Crystal-Structure Determination. *Acta Crystallogr. A Found. Adv.* **2015**, *71* (1), 3–8.

(2) Sheldrick, G. M. Crystal Structure Refinement with *SHELXL*. *Acta Crystallogr. C Struct. Chem.* **2015**, *71* (1), 3–8.

(3) Spek, A. L. Structure Validation in Chemical Crystallography. *Acta Crystallogr. D Biol. Crystallogr.* **2009**, *65* (2), 148–155.

(4) Dolomanov, O. V.; Bourhis, L. J.; Gildea, R. J.; Howard, J. A. K.; Puschmann, H. *OLEX2*: A Complete Structure Solution, Refinement and Analysis Program. *J. Appl. Crystallogr.* **2009**, *42* (2), 339–341.

(5) Bajada, M. A.; Di Liberto, G.; Tosoni, S.; Ruta, V.; Mino, L.; Allasia, N.; Sivo, A.; Pacchioni, G.; Vilé, G. Light-Driven C–O Coupling of Carboxylic Acids and Alkyl Halides over a Ni Single-Atom Catalyst. *Nat. Synth.* **2023**, *2* (11), 1092–1103.

(6) Ullah, M.; Lv, H.; Liu, Z.; Bai, X.; Chen, J.; Zhang, Y.; Wang, J.; Sun, B.; Li, L.; Shi, K. Rational Fabrication of a g-C_3N_4/NiO Hierarchical Nanocomposite with a Large Surface Area for the Effective Detection of NO_2 Gas at Room Temperature. *Appl. Surf. Sci.* **2021**, *550*, 149368.

(7) Liu, J.; Zou, Y.; Cruz, D.; Savateev, A.; Antonietti, M.; Vilé, G. Ligand–Metal Charge Transfer Induced *via* Adjustment of Textural Properties Controls the Performance of Single-Atom Catalysts during Photocatalytic Degradation. *ACS Appl. Mater. Interfaces* **2021**, *13* (22), 25858–25867.

(8) Nizovtsev, A. S. Interaction of Carbon Monoxide with Transition Metal Phthalocyanines. *J. Struct. Chem.* **2023**, *64* (7), 1275–1282.
